# Externalized decondensed neutrophil chromatin occludes pancreatic ducts and drives pancreatitis

**DOI:** 10.1038/ncomms10973

**Published:** 2016-03-11

**Authors:** Moritz Leppkes, Christian Maueröder, Sebastian Hirth, Stefanie Nowecki, Claudia Günther, Ulrike Billmeier, Susanne Paulus, Mona Biermann, Luis E. Munoz, Markus Hoffmann, Dane Wildner, Andrew L. Croxford, Ari Waisman, Kerri Mowen, Dieter E. Jenne, Veit Krenn, Julia Mayerle, Markus M. Lerch, Georg Schett, Stefan Wirtz, Markus F. Neurath, Martin Herrmann, Christoph Becker

**Affiliations:** 1 Department of Medicine 1, University Clinics Erlangen, University of Erlangen-Nuremberg, 91054 Erlangen, Germany; 2 Department of Medicine 3, University Clinics Erlangen, University of Erlangen-Nuremberg, 91054 Erlangen, Germany; 3 Institute of Molecular Medicine, University Medicine of Johannes Gutenberg University, 55131 Mainz, Germany; 4 Department of Chemical Physiology, Scripps Institute, La Jolla, California 92037, USA; 5 Department of Immunology and Microbial Sciences, Scripps Institute, La Jolla, California 92037, USA; 6 Institute of Lung Biology and Disease, Comprehensive Pneumology Center, 81377 Munich, Germany; 7 Department of Pathology, MVZ of Pathology, 54296 Trier, Germany; 8 Department of Medicine A, University Medicine Greifswald, 17475 Greifswald, Germany

## Abstract

Ductal occlusion has been postulated to precipitate focal pancreatic inflammation, while the nature of the primary occluding agents has remained elusive. Neutrophils make use of histone citrullination by peptidyl arginine deiminase-4 (PADI4) in contact to particulate agents to extrude decondensed chromatin as neutrophil extracellular traps (NETs). In high cellular density, NETs form macroscopically visible aggregates. Here we show that such aggregates form inside pancreatic ducts in humans and mice occluding pancreatic ducts and thereby driving pancreatic inflammation. Experimental models indicate that PADI4 is critical for intraductal aggregate formation and that PADI4-deficiency abrogates disease progression. Mechanistically, we identify the pancreatic juice as a strong instigator of neutrophil chromatin extrusion. Characteristic single components of pancreatic juice, such as bicarbonate ions and calcium carbonate crystals, induce aggregated NET formation. Ductal occlusion by aggregated NETs emerges as a pathomechanism with relevance in a plethora of inflammatory conditions involving secretory ducts.

## Abstract (web summary)

Pancreatitis often develops as a consequence of ductal obstruction. Here, the authors show that bicarbonate ions initiate the release of neutrophil extracellular traps (NETs) that form pancreatic ductal aggregates and occlude the ducts, thereby driving pancreatitis in mice and humans.

## Graphical abstract

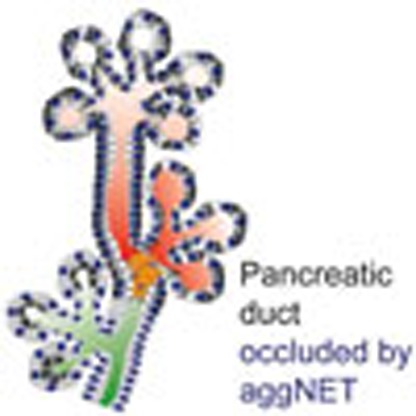

Inflammatory disorders of the pancreas present a broad spectrum of severity ranging from mild oedematous pancreatitis to life-threatening severe acute pancreatitis with fundamental differences in pathogenesis depending on the underlying causative factors^1^. If the underlying instigators of inflammation remain active, the exocrine gland undergoes fibroinflammatory remodelling typical of chronic pancreatitis. While severe acute pancreatitis may cause lethality based on systemic complications of disease, a chronic course of disease may result in a debilitating disorder leading to chronic pain, maldigestion and severe weight loss^2^. Histopathologically, chronic pancreatitis is characterized by parenchymal remodelling, which may display a focal distribution, while adjacent exocrine tissue may remain intact during the course of the disease. The focal nature is a well-known, yet less understood feature of pancreatic inflammation^3^,^4^, recapitulated in some experimental models of this disease^5^. Chronic pancreatitis may arise from diverse aetiologies recapitulated by the TIGAR-O concept^6^, including toxic–metabolic, idiopathic, genetic, autoimmune, recurrent and severe acute pancreatitis, and obstructive causes^7^. Due to the common ductal system, biliary disease may cause acute pancreatitis due to ductal prepapillary impaction by small gallstones or so-called biliary sludge^8^,^9^. Cholangitis as evidenced by increased liver function tests is the most important trigger for medical intervention to resolve ductal obstruction^10^. The time frame of ductal occlusion strongly determines severity of pancreatitis^11^. Neutrophils are part of the inflammatory infiltrate in acute to chronic pancreatitis, yet are less present in samples of postinflammatory fibrosis, indicating their importance in earlier phases of pancreatitis^7^. Granulocytic epithelial lesions containing intra- and periductal granulocyte aggregates have been postulated as a pathognomonic hallmark of autoimmune pancreatitis type 2 (ref. 12), a newly identified, rarely diagnosed disease.

We hypothesized that the formation of intraductal neutrophil-rich aggregates and consequent ductal occlusion orchestrates the focal appearance of pancreatitis.

Recently, the molecular mechanisms of neutrophil aggregation have been further elucidated. Neutrophils make use of reactive oxygen species and histone citrullination by peptidyl arginine deiminase-4 (PADI4)^13^,^14^,^15^ in contact to biochemical and particulate stimuli. These processes then lead to the extrusion of decondensed chromatin as DNase-sensitive neutrophil extracellular traps (NETs)^16^,^17^ endowed with functional neutrophil serine proteases^18^. Extracellular chromatin of NETs supports the aggregation of viable, necrotic and apoptotic cells as well as particulate matter (crystals and microbes). In addition, extracellular chromatin of NETs has been shown to support platelet aggregation in blood clot formation^19^. Aggregates of NETs have been previously shown to form macroscopically visible structures: these aggregated NETs (aggNETs) displayed anti-inflammatory properties due to proteolysis of cytokines and chemokines in gout^20^.

We observed that neutrophils may enter the lumen of biliopancreatic ducts under inflammatory conditions and form aggregates of NETs, which then hamper secretory flow, and thereby drive focal pancreatitis and parenchymal remodelling depending on PADI4. Cellular changes induced by components of the pancreatic juice, such as elevated levels of bicarbonate, support PADI4 activity and induce H3cit^+^ NET formation.

## Results

### Interleukin-17A delivery induces pancreatitis

**Figure 1:**
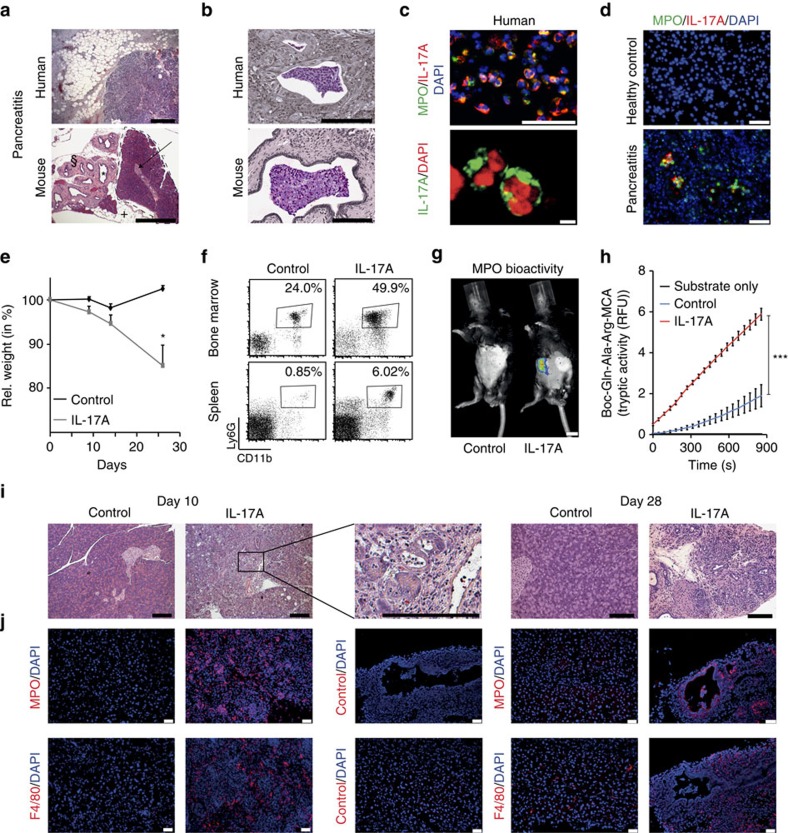
Delivery of IL-17A induces pancreatitis with intraductal neutrophils. (**a**,**b**) Haematoxylin and eosin staining of tissue sections obtained from human chronic pancreatitis (**a**), malignancy-related pancreatic inflammation (**b**) and murine pancreatitis induced by IL-17A delivery. (specific features are marked in the lower picture: +, fatty degeneration; §, fibrotic stroma; *, pseudotubular complex; dotted line and arrow, healthy area). (**a**) Note the focal nature of the disease with remodelled adjacent to intact glandular tissue (representative of *n*=13 (human) and *n*⩾15 (mouse); bar, 500 μm); (**b**) note cell-containing aggregates (coloured) inside pancreatic ducts (grey scale) in murine (15/15) and human samples (0/5 benign chronic pancreatitis, 3/8 malignancy associated). (**c**) Top: cells with segmented nuclei inside intraductal aggregates in human pancreatitis showed dual labelling for MPO and IL-17A (3/3); bottom: a close-up shows IL-17A immunoreactivity in a human granulocyte with a typical segmented nucleus (bar, 5 μm). (**d**) In IL-17A-induced pancreatitis, IL-17A^+^MPO^+^ aggregates were observed in the lumen of acini and ducts. No MPO- or IL-17A-positive cells were observed in control tissue (*n*=4 per group). (**e**) Note wasting in IL-17A-treated mice (*n*=8 per group, mean+s.e.m.). (**f**) Analysis by flow cytometry of bone marrow and spleen cells demonstrated IL-17A-enforced granulopoesis and neutrophil mobilization (*n*⩾15 per group). (**g**) Luminol bioluminescence imaging showed MPO activity in the upper abdomen of IL-17A-treated mice only, projected on the mesenteric part of the pancreas (*n*=4 per group; bars, 1 cm). (**h**) Tryptic activity of pancreas homogenates was fluorometrically assessed and showed a significant increase after IL-17A delivery as compared with control (two independent experiments, *n*=6 per group). (**i**,**j**) Analyses at day 10 and day 28 after IL-17A delivery demonstrated leukocyte infiltration at day 10, diminishing over time and a progressive remodelling of the pancreas. The control vector does not induce morphologic changes (*n*⩾8 per time point and group). (**i**) Immunohistochemistry reveals infiltration of neutrophils and macrophages into the pancreas 10 days after IL-17A delivery (*n*⩾10 per group). MPO^+^ cells displays patchy aggregation. After 28 days, MPO^+^ aggregates were found inside a pancreatic duct and in the periductal area. The aggregates are negative for F4/80, which is confined to myeloid cells throughout the fibroinflammatory stroma. Black scale bars, 200 μm; white scale bars, 50 μm, unless stated otherwise. (*P<0.05, ***P<0.001, Student's *t*-test).

We initially detected cellular aggregates containing neutrophils inside pancreatic ducts in both human and murine sections of inflamed pancreatic tissue, which showed a focal distribution of inflammation (Fig. 1a,b). Interestingly, intraductal aggregates contained interleukin-17A (IL-17A) in both human and murine samples (Fig. 1c,d)^21^. IL-17A expression was detected in MPO^+^ cells with segmented nuclei, characteristic of neutrophil granulocytes (Fig. 1c). IL-17A is a critical cytokine regulating the granulocyte pool^22^. Besides other sources^23^,^24^, it has previously gained attention as signature cytokine of the Th17 lineage^25^. A recent study has renewed the interest in IL-17A, as it displays proinflammatory and protumorigenic activities in pancreatic malignancy-associated inflammation^26^. Therefore, we were interested in the *in vivo* effects of IL-17A and employed two modes of its systemic delivery using transgenic and vector-based approaches (Supplementary Fig. 1A–D). Surprisingly, delivery of IL-17A induced a progressive wasting disease in mice (Fig. 1e; Supplementary Fig. 1E)^27^. IL-17A challenge induced granulopoesis, and the mobilization of CD11b^+^Ly6G^+^ neutrophils was markedly enhanced (Fig. 1f). Moreover, bioluminescence imaging revealed substantial myeloperoxidase (MPO) activity in the upper abdomen, projected on the mesenteric area of the rodent pancreas (Fig. 1g). Tryptic activity of the pancreas homogenate was significantly elevated (Fig. 1h), indicating a premature activation of digestive zymogens in the inflamed pancreas. The histological analysis revealed striking myeloid inflammatory infiltrations and morphologic alterations of the pancreas after IL-17A delivery only (Fig. 1i,j). Of note, other organs displayed minor leukocyte infiltrations in the absence of overt pathology (Supplementary Fig. 1F–H). Intestinal permeability was not increased by either IL-17A delivery or control vector treatment (Supplementary Fig. 1G). Five days after birth, IL-17A-expressing mice showed regular pancreas development and displayed no signs of inflammation (Supplementary Fig. 2A). After weaning (postnatal day 28), a strong inflammatory infiltration concomitant with progressive acinar destruction, fatty degeneration and pseudotubular complex formation was observed (Fig. 1i; Supplementary Fig. 2A), while remnants of regular tissue architecture were noted. Interestingly, IL-17A expression did not lead to a marked increase in serum amylase and lipase activities (Supplementary Fig. 2B), typical of acute pancreatitis. Trichrome staining displayed pancreatic fibrosis in affected mice corroborated by a gene expression profile indicative of fibrosis such as changes in the expression of matrix metalloproteases and *tgfb* (Supplementary Fig. 2C,E)^28^. Moreover, fibrotic remodelling was evident by the expansion of mesenchymal cells in IL-17A-induced pancreatitis as indicated by vimentin and α-smooth muscle actin immunohistochemistry (Supplementary Fig. 2F)^29^. Mice that lost weight due to IL-17A delivery showed regular fasting glucose levels, thereby excluding overt diabetes in these mice (Supplementary Fig. 2D). Yet, faecal triglycerides were strongly increased after 24 h of a high-fat diet indicative of exocrine pancreatic insufficiency (Supplementary Fig. 2G). Infiltrating lymphocytes in the inflamed pancreas were rare and mostly identified as non-T cells (Supplementary Fig. 3A). Functionally, the absence of T and B lymphocytes in Rag1^−/−^ mice did not alter IL-17A-induced granulopoesis, neutrophil mobilization and aggregation in the pancreas (Supplementary Fig. 3A–D). IL-17A-induced pancreatitis thereby constitutes an inflammatory process not instigated by the adaptive immune system. Lymphocytes may, however, constitute a cellular source of IL-17A, in other models of pancreatitis or human pathophysiology.

It has been proposed that IL-17A induces weak biological responses in various cell types, but crucially augments the effects of the proinflammatory cytokines tumour necrosis factor α (TNFα), IL-1β or IL-6 (ref. 30). Indeed, after IL-17A delivery, we detected elevated levels of these cytokines in inflamed pancreata and IL-6 in the serum (Supplementary Fig. 4A,B). We observed that both pancreatic acinar and stromal cells are cellular targets of IL-17A: expression of the known IL-17A target gene *Cxcl5* was induced by this cytokine in isolated cultures of both tissue resident cell populations *in vitro* (Supplementary Fig. 4C). Moreover, IL-1β and IL-17A synergistically induced expression of this chemokine (Supplementary Fig. 4D). To elucidate the functional contribution of these mediators on pancreatic neutrophil aggregate formation *in vivo*, we took advantage of IL1 receptor-1-deficient (*IL1R1*^*−/−*^), TNF receptor-1+2-deficient (*TNFR1+2*^*−/−*^) and IL-6-deficient (*IL6*^*−/−*^) mice^31^. IL-17A delivery in these strains revealed that neither IL-6 nor signalling via these TNF or IL-1 receptors were required for the development of pancreatitis: all mice tested developed neutrophil aggregates and pancreatitis in response to IL-17A in a similar manner (Supplementary Fig. 4E,F). The ectopic expression of IL-17A in this model might bypass the need of these proinflammatory mediators in pancreatic inflammation. In other models of pancreatitis, the crucial effects of these mediators have been observed^31^,^32^,^33^. Each single mediator may yet be subject to redundancy with other NF-κB-activating pathways.

### IL-17A-induced pancreatitis depends on PADI4 in neutrophils

**Figure 2:**
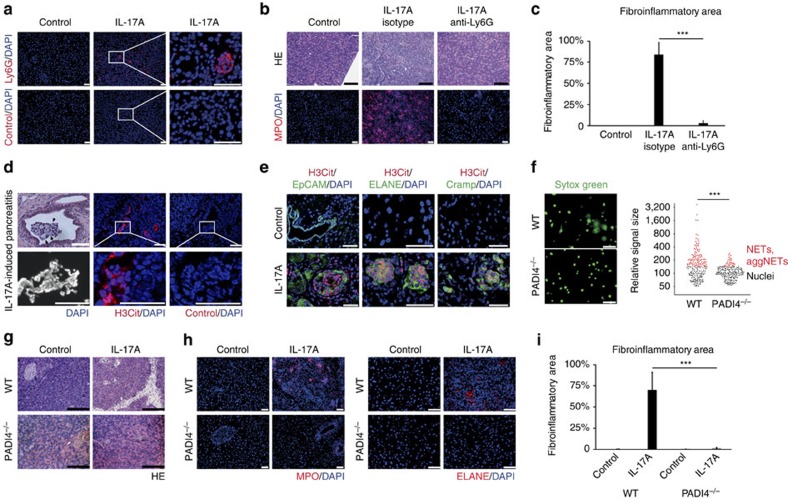
IL-17A-induced pancreatitis is driven by PADI4-dependent neutrophil aggregates. (**a**) Ly6G immunohistochemistry detected neutrophil granulocyte aggregates in the pancreas after IL-17A challenge only. (**b**,**c**) Mice treated with IL-17A delivery or control were injected with a neutrophil-depleting anti-Ly6G antibody (1A8) or isotype control (2A3) (three independent experiments with *n*=12 per group, ***P<0.001 (analysis of variance, Tukey honest significant difference). (**b**) Haematoxylin and eosin (H&E) staining (top) and MPO immunofluorescence (bottom) showed that IL-17A-induced pancreatitis depends on Ly6G^+^ granulocytes. (**c**) The area subject to fibroinflammatory remodelling was strongly reduced by anti-Ly6G treatment. The isotype antibody was without protective effect. (mean+s.e.m.). (**d**) A close-up of an aggregate after IL-17A delivery displayed neutrophils bound together by amorphous haematoxylin-stained fibres (day 28; top left). 4,6-Diamidino-2-phenylindole (DAPI) staining revealed DNA configured in non-nuclear morphology inside these ducts (bottom left). Immunohistochemistry of citrullinated histone H3 (H3cit) was detected on extracellular DNA (centre, right: staining control). (**e**) EpCAM/H3cit co-staining revealed aggregates formed in pancreatic ducts and metaplastic acini (day 10 after IL-17A delivery). Co-staining of neutrophil elastase (ELANE), Cramp and H3cit showed ELANE and Cramp on intraductal aggregates with PADI4 activity, even in areas, in which extracellular DNA was below detection level. The cellular density of intact neutrophils inside aggregates was increased at day 10 as compared with day 28, precluding a closer examination of non-nuclear DNA. H3cit was also detectable inside granulocyte nuclei at this time point. (**f**) Thioglycolate-elicited neutrophils from wild-type and PADI4^−/−^ mice were stimulated with lipopolysaccharide (100 ng ml^−1^), and chromatin morphology was determined by Sytox Green fluorescence. Wild-type cells showed an increased size of the area covered by DNA and bizarrely configured DNA tangles, reflecting chromatin decondensation. Chromatin appeared more condensed in PADI4^−/−^ neutrophils (>3 independent experiments). (**g**–**i**) Wild-type and PADI4^−/−^ mice were treated with IL-17A or a mock control vector. (**g**) H&E-staining revealed no pancreatitis development in PADI4^−/−^ mice in response to IL-17A. (**h**) MPO and ELANE immunohistochemistry showed only few MPO^+^ or ELANE^+^ cells in the pancreata of PADI4^−/−^ IL-17A-challenged mice. In wild-type controls, patchy neutrophil accumulation was noted all-over the section (two independent experiments with a total of *n*⩾12 per group. (**i**) The area affected by fibroinflammatory remodelling was strongly diminished in PADI4^−/−^ mice (mean+s.e.m.; black scale bars, 200 μm; white scale bars, 50 μm, unless stated otherwise. ***P<0.001, Student's *t*-test).

We observed Ly6G-expressing neutrophil granulocytes to be the main infiltrating immune cell population in IL-17A-induced pancreatitis (Fig. 2a; Supplementary Fig. 5A). Therefore, we tested the effects of repetitive circulatory neutrophil depletion using anti-Ly6G on the development of IL-17A-induced pancreatitis to functionally assess the role of neutrophil aggregates *in vivo*. Anti-Ly6G strongly reduced the fraction of granulocytes (CD11b^+^SSC^hi^) in the circulation, even in the context of IL-17A-enhanced granulopoiesis (Supplementary Fig. 5B). Strikingly, repetitive anti-Ly6G treatment effectively precluded neutrophil infiltration to the pancreas^34^ and abrogated the development of pancreatitis (Fig. 2b,c). These findings are in line with previous observations in other models of pancreatitis^35^,^36^,^37^,^38^: while the acinar architecture of mice treated with isotype antibodies was massively compromised after IL-17A delivery, anti-Ly6G-treated mice demonstrated regular acini and only minimal inflammation. Moreover, isotype-treated mice showed extensive neutrophil aggregation in their pancreata, whereas only single MPO^+^ cells were observed around pancreatic ducts of anti-Ly6G-treated mice (Fig. 2b). Importantly, in those mice, the extent of cell death as assessed by TUNEL (TdT-mediated dUTP-biotin nick-end labelling) and cleaved caspase 3 immunohistochemistry was strongly reduced in pancreatic tissue sections devoid of neutrophils (Supplementary Fig. 5C,D). We thereby excluded a direct cytotoxic effect of IL-17A on the acinar cell population.

Neutrophil aggregation has been shown to be supported by extracellular DNA^20^. We hypothesized that chromatin decondensation and extracellular deposition as in NETs might also contribute to the aggregate formation in pancreatic ducts. Indeed, histological studies of intraductal pancreatic aggregates revealed a substantial amount of DNA in structures, lacking the distinct morphologies of nuclei (Fig. 2d). We therefore denominated these intraductal aggregates as aggNETs. Neutrophil chromatin decondensation is regulated by a charge-dependent loss of histone DNA affinity due to histone arginine citrullination by PADI4 (ref. 14). Using immunopositivity for citrullinated histone H3 (H3cit) as a surrogate of PADI activity revealed citrullination of histone H3 in these aggNETs. H3cit was found both in intraductal granulocytic nuclei and was strongly detected co-localizing with extranuclear DNA in the intraluminal space directly adjacent to intact granulocytes. In contrast, in healthy control pancreas, H3cit was not detectable (Fig. 2d, e). Co-staining with epithelial cell adhesion molecule (EpCAM) revealed that H3cit was predominantly observed inside pancreatic ducts and in the lumen of acini undergoing ductal metaplasia (Fig. 2e), as indicated by increased acinar EpCAM staining. In addition to H3cit positivity in aggNETs, neutrophil-specific elastase (ELANE) and the cathelicidin-related antimicrobial peptide (Cramp) were detected in the extracellular intraductal space.

To functionally assess the contribution of chromatin decondensation/aggregation *in vivo*, we made use of *PADI4*^*−/−*^ mice, previously shown to be deficient in neutrophil histone citrullination and NET formation (Fig. 2f)^15^. IL-17A-enforced granulopoesis and neutrophil mobilization from bone marrow was similar in wild-type and *PADI4*^*−/−*^ mice (Supplementary Fig. 6A,B). In addition, IL-17A target genes were also elevated in pancreatic tissue from *PADI4*^−/−^ mice, indicating functional IL-17A signal transduction in pancreatic cells (Supplementary Fig. 6C). Strikingly, *PADI4* deficiency strongly protected mice from the development of IL-17A-induced pancreatitis (Fig. 2g–i): signs of fibroinflammatory remodelling such as pseudotubular complex formation and acinar cell loss were absent. Only single MPO^+^ cells were observed in the periductal area of these pancreata, whereas MPO^+^ cells were abundantly present and aggregated throughout the wild-type pancreata (Fig. 2h). Importantly, neutrophils from *PADI4*-deficient mice were recruited as efficiently as wild-type neutrophils to the peritoneal cavity excluding a general cell-intrinsic defect in transendothelial migration and tissue recruitment (Supplementary Fig. 6D)^39^. We conclude that PADI4-mediated arginine citrullination in the context of aggNET formation vitally contributes to IL-17A-induced pancreatitis.

To assess the role of PADI4 in an independent model, we first made use of caerulein-induced experimental acute pancreatitis (Supplementary Fig. 7A–H), which progresses to chronic pancreatitis when the protocol is performed repeatedly (Supplementary Fig. 7I–K)^28^. This model is mainly driven by stimulus-induced acinar cell death (‘necrosis–fibrosis concept')^40^, while neutrophils may alter the disease course subordinately^33^,^37^. Neutrophil accumulation in caerulein-induced pancreatitis is fundamentally different from neutrophil accumulation in intraductal aggNETs in the IL-17A-induced model. Specifically, no intraductal aggNETs can be identified in this model. As expected, and in contrast to the IL-17A challenge model, intraductal aggNETs containing H3cit were absent in caerulein-induced pancreatitis (Supplementary Fig. 7C). Consequently, deficiency of PADI4 did not significantly alter the disease course in this model: histological analyses of pancreatic tissue showed an indistinguishable severity regarding acinar damage and immune cell infiltration (Supplementary Fig. 7A,D,E). Amylase and lipase activities were elevated to the same extent in both wild-type and *PADI4*-deficient mice (Supplementary Fig. 7B). In addition, both groups showed lung damage, and alveolar neutrophil infiltration in response to caerulein was equal (Supplementary Fig. 7F–H). Furthermore, when caerulein injections were performed repeatedly to induce a chronic course of disease, acinar cell loss, pancreatic fibrosis and pseudotubular complex formation occurred in both wild-type and *PADI4*-deficient mice to a similar extent (Supplementary Fig. 7I–K).

In summary, both acute caerulein-induced pancreatitis and its progression to chronicity occur independently of PADI4. This is in line with the concept of direct caerulein-induced acinar cytotoxicity in this model, the absence of intraductal aggNETs and with reports using myeloperoxidase-deficient mice in this model^37^, which were equally affected of caerulein-induced pancreatitis and for which a defective NET formation has also been described^41^.

### AggNETs in benign- and malignancy-associated pancreatitis

**Figure 3:**
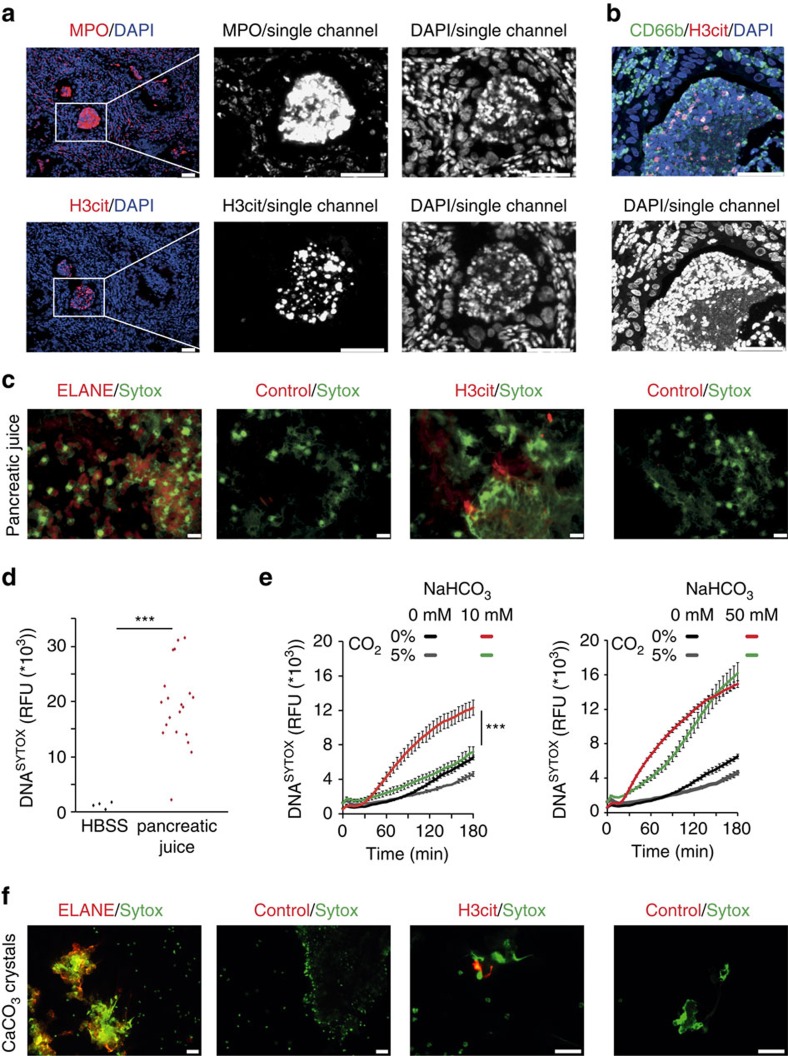
Components of pancreatic juice facilitate externalization of decondensed neutrophil chromatin. (**a**) Immunofluorescence of myeloperoxidase (MPO) and citrullinated histone H3 (H3cit) revealed intraductal neutrophil aggregates in human pancreatic inflammation (immunopositivity rates of samples: malignancy-related pancreatitis (3/8) and benign chronic (0/5) pancreatitis, respectively); single channels are provided to appreciate low-intensity extracellular DNA between intact neutrophils. (**b**) Intraductal aggregates display CD66b^+^ cells and co-labelling of H3cit and extracellular DNA (3/3). (**c**) Cytospins from fresh patient-derived pancreatic fluid punctates of patients with benign pancreatitis revealed DNA webs positive for (left) neutrophil elastase (ELANE) and (right) H3cit with the respective staining control (right column; *n*=3/3). (**d**,**e**) Freshly isolated human blood neutrophils were cultured in human pancreatic juice or isotonic HBSS-based buffers containing varying amounts of NaHCO_3_, as indicated. DNA in the cell culture was quantified with a Sytox Green fluorimetric assay detecting extracellular DNA and chromatin of permeabilized cells only (DNA^SYTOX^). (**d**) Quantitative assessment indicated strong increases in DNA^SYTOX^ of human neutrophil cultures in response to human pancreatic juice (*n*=20; 3 h of stimulation). (**e**) NaHCO_3_ dose dependently raises DNA^SYTOX^ as compared with NaHCO_3_-free conditions. This effect was facilitated by ambient pCO_2_ levels. Five percent of CO_2_ was able to inhibit DNA detection induced by 10 mM NaHCO_3_, yet failed to inhibit DNA detection at higher concentrations of NaHCO_3_ (50 mM; *n*=3 independent experiments, mean+s.e.m.). (**f**) Freshly isolated human blood neutrophils grown on coverslips developed decondensed chromatin and aggregates positive for (left) neutrophil elastase and citrullinated histones (right) in response to calcium carbonate crystals (5 mg ml^−1^, ⩾4 independent experiments). (All white scale bars, 50 μm. *P<0.05, **P<0.01, ***P<0.001, Student's *t*-test.)

To assess the importance of aggNETs to human pancreatic diseases, we next studied samples from patients with benign- or malignancy-related pancreatitis using both histological tissue sections and endoscopy-derived samples of pancreatic juice. Several samples of inflamed human pancreatic tissue displayed MPO- and H3cit-positive intraductal neutrophil aggregates (Fig. 3a). These intraductal aggNETs contained CD66b^+^ granulocytes and large amounts of extracellular DNA, as demonstrated by histochemistry (Fig. 3b). Of note, the signal intensity of spread extracellular chromatin was markedly lower than that of intact nuclei. These aggNETs were broadly detectable in multiple sections of malignancy-related pancreatic inflammation only (3/8 specimens). As expected, only residual pancreatic fibrosis devoid of any potentially causative inflammatory infiltrate was observed in the surgical specimens of benign idiopathic chronic pancreatitis. Noteworthy, a specific occluding agent in the proximal ductal tree, such as an aggNET, may not be evident in random sections of distant parts of the organ. Therefore, we resorted to samples of pancreatic juice of patients suffering from pancreatitis to specifically analyse the ductal content. AggNETs were readily detected in 4/10 and in 6/10 samples of pancreatic juice of patients with benign- and malignancy-associated pancreatitis, respectively, as well as in 3/3 punctates of dilated ducts and pancreatic pseudocysts. Confirmatory immunocytochemistry detected H3cit and ELANE in all three samples of pancreatic punctates (Fig. 3c). This implies the importance of aggNETs in the pathophysiology of pancreatitis in general, not limited to malignancy.

### Components of pancreatic juice facilitate aggNET formation

**Figure 4:**
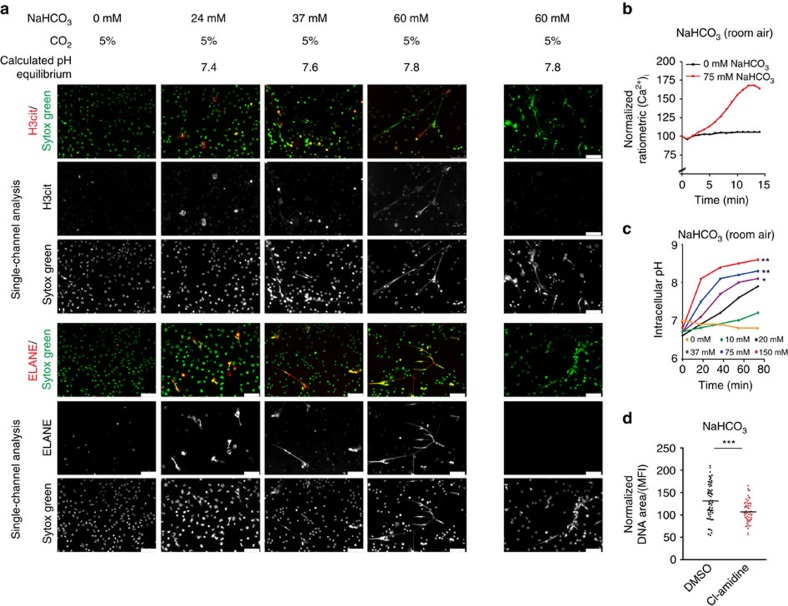
NaHCO_3_-induced cellular changes support PADI4 activity. (**a**) Freshly isolated human neutrophils from the peripheral blood of healthy donors were cultured on glass coverslips for 120 min at 37 °C/5% CO_2_ and subjected to isotonic HBSS media containing different concentrations of HCO_3_^−^. The calculated pH equilibrium of the bicarbonate–CO_2_ buffer system of these media at 37 °C/5% CO_2_ is specified. Immunocytochemistry of H3cit (top) and neutrophil elastase (bottom), and the respective DNA^SYTOX^ counterstain in both overlay as well as single-channel analyses are provided. Please note the absence of H3cit^+^ ELANE^+^ extracellular chromatin in the absence of NaHCO_3_. The presence of NaHCO_3_ in the media leads to marked increases in H3cit^+^ ELANE^+^ extracellular chromatin reminiscent of NETs (representative pictures of one of three independent experiments are shown). (**b**) Ratiometric determination of the cytosolic Ca^2+^ concentration revealed the elevation of [Ca^2+^]_I_ induced by 75 mM NaHCO_3_ (*n*=4 independent experiments). (**c**) The intracellular pH of human neutrophils was measured by flow cytometry employing SNARF as pH-sensitive dye under ambient pCO_2_. Note the time- and bicarbonate-concentration-dependent increase of the cytoplasmic pH (*n*=3 independent experiments, mean+s.e.m.). (**d**) Chromatin externalization by 50 mM bicarbonate was induced in human granulocytes from healthy donors in the presence or absence of the PADI inhibitor Cl-amidine (1 mM) and images of propidium iodide fluorescence as in Supplementary Fig. 8H were morphometrically analysed. The nuclear decondensation is reflected by an increased ratio of chromatin area to mean fluorescence intensity (MFI) in flow cytometry. The data are normalized to 100 for an average healthy nucleus. Note the nuclear decondensation induced by bicarbonate, which was reduced in the presence of Cl-amidine (⩾3 independent experiments). (All white scale bars, 50 μm. *P<0.05, **P<0.01, ***P<0.001, Student's *t*-test).

Next, we addressed possible instigators of aggNET formation in pancreatic ducts. These ducts contain liquid, which is called pancreatic juice. Neutrophils from healthy donors cultured in pancreatic juice derived from chronic pancreatitis extruded decondensed chromatin to the extracellular space as demonstrated by live-cell video microscopy (Supplementary Fig. 9A; Supplementary Movie 1). Sytox Green detects extracellular DNA and DNA of permeabilized cells only (DNA^SYTOX^). Quantitative fluorimetric studies of neutrophils cultured in human pancreatic juice (chronic pancreatitis, pancreatic cancer) showed strong increases in DNA^SYTOX^ (Fig. 3d). We then studied which components of pancreatic juice contribute to increases in DNA^SYTOX^ in neutrophil cell cultures. We first examined the ionic composition of pancreatic juice. Pancreatic juice most strongly differs from serum with regard to its mildly alkaline pH and a bicarbonate concentration of up to 150 mM (Supplementary Fig. 8; Supplementary Table 1). Bicarbonate is present in the juice of both healthy individuals and affected patients. Its concentration increases strongly after meals to neutralize the gastric content in the small intestine^42^. We speculated that bicarbonate might contribute to aggNET formation in pancreatic juice. Indeed, video microscopy revealed neutrophil chromatin extrusion (Supplementary Fig. 9B–D; Supplementary Movies 2–5) in response to NaHCO_3_. Macroscopically visible aggregates were formed *in vitro* (Supplementary Fig. 8A), which were immunopositive for neutrophil elastase and H3cit. Quantitative fluorimetry showed significant dose-dependent increases in DNA^SYTOX^ in response to bicarbonate (Fig. 3e; Supplementary Fig. 8C). These increases in DNA^SYTOX^ were facilitated by the open buffer system of room air providing ambient CO_2_ (0.4%). Increases in DNA^SYTOX^ at low concentrations of NaHCO_3_ were inhibited by serum concentrations of 5% CO_2_ (Fig. 3e). Yet, NaHCO_3_ concentrations above the serum level of 24 mM induced marked increases in DNA^SYTOX^ extrusion even in the presence of 5% CO_2_ (Figs 3e and 4). The overall increase in DNA^SYTOX^ induced by NaHCO_3_ was in the same range as induced by pancreatic juice or phorbol-12-myristate-13-acetate (PMA), respectively (Supplementary Fig. 8D). A gaseous loss of osmotic CO_2_ equivalents in bicarbonate-containing buffers could induce changes in buffer osmolality during the experiments. Theoretically, the possible maximal loss of CO_2_ equivalents in a buffer containing 50 mM NaHCO_3_ could reduce the osmolality by 50 mOsm kg^−1^. Carbonate-free buffers with reduced osmolality in this range did not markedly increase DNA^SYTOX^ (Supplementary Fig. 8E). Thus, osmolality changes in this range cannot account for the strong increases in DNA^SYTOX^ observed. Bicarbonate-buffer-induced increases in DNA^SYTOX^ were dose dependently inhibited by the carbonic anhydrase inhibitor acetazolamide (Supplementary Fig. 8F), further implicating the involvement of the bicarbonate–CO_2_ axis in this process. Acetazolamide also significantly blocked increases in DNA^SYTOX^ induced by pancreatic juice devoid of tryptic activity (Supplementary Fig. 8F). While patients with chronic pancreatitis may actually show diminished maximal levels of 80 mM HCO_3_^−^ in pancreatic juice, these levels are still far above the level tolerable to neutrophils *in vitro* (Fig. 3f; Supplementary Fig. 8C). Pancreatic juice is supersaturated with both calcium and carbonate ions^43^. Interestingly, we observed chromatin extrusion of neutrophils and H3cit, and ELANE-positive aggNETs also in response to CaCO_3_ crystals, as found in pancreatic calcifications (Fig. 3f)^44^. This shows that aggNET formation in pancreatic ducts can be induced by multiple stimuli. It furthermore adds another type of crystals to the list of NET inducers in diverse disease conditions^20^: recently, cholesterol crystal-induced NETosis has been identified as a major driver of atherosclerotic plaque formation^45^.

### Bicarbonate-induced cellular changes support PADI4 activity

Importantly, we detected neutrophil elastase and PADI4 activity (H3cit) in bicarbonate-induced aggNETs and a specific co-localization of H3cit and elastase to DNA in neutrophil-derived extracellular chromatin reminiscent of NETs (Fig. 4a). PADI4 activity is strongly calcium-dependent^46^. Furthermore, maximal activity of this enzyme has been determined at pH 7.6–8 (ref. 47). Bicarbonate stimulation strongly increased the cytosolic calcium concentration (Fig. 4b) of neutrophils, as determined by Fura-2/Fluo-3 ratios. Moreover, we found that bicarbonate dose dependently alkalinizes the cytoplasm of neutrophils (Fig. 4c; Supplementary Fig. 9C; Supplementary Movie 3). In flow cytometric measurements, the intracellular pH rose up to 8.2 after incubation in a buffer containing 37 mM sodium bicarbonate. However, this does not truly reflect the *in vivo* situation, since flow cytometry did not allow CO_2_ control in the samples. According to the Henderson–Hasselbalch equation, the calculated intracellular pH equilibrium of 37 mM NaHCO_3_/5% CO_2_ is 7.6 at 37 °C. Of note, carbonate-free alkaline phosphate buffers did not induce chromatin extrusion at these levels, indicating that bicarbonate ions have an intrinsic property not reflected solely by the change of the pH (Supplementary Fig. 8G). It should be mentioned, that non-physiological bicarbonate-free phosphate buffers equilibrated to a pH as high as 8.4 also increased DNA^SYTOX^ in our assays, but not below the threshold of pH 8.4 (Supplementary Fig. 8G). It should be noted that we deliberately excluded synthetic buffers such as HEPES in these experiments to better reflect the *in vivo* setting inside the pancreatic ducts.

Thus, bicarbonate stimulation of neutrophils provides two major prerequisites facilitating PADI4 activity: an increase of both intracellular pH and cytosolic calcium. Functionally, inhibition of PADI significantly reduced chromatin decondensation in bicarbonate-stimulated human neutrophils (Fig. 4d; Supplementary Fig. 8H,I).

It has been shown that NETs are sensitive to DNase^17^. Protective effects of DNase-I in models of acute pancreatitis were recently described and also attributed to NET digestion^48^. Indeed, DNase easily digested small extracellular chromatin fibres of bicarbonate- and CaCO_3_-induced aggNETs, while the core of aggNETs was more resistant (Supplementary Fig. 8J).

### AggNETs occlude pancreatic ducts and drive pancreatitis

**Figure 5:**
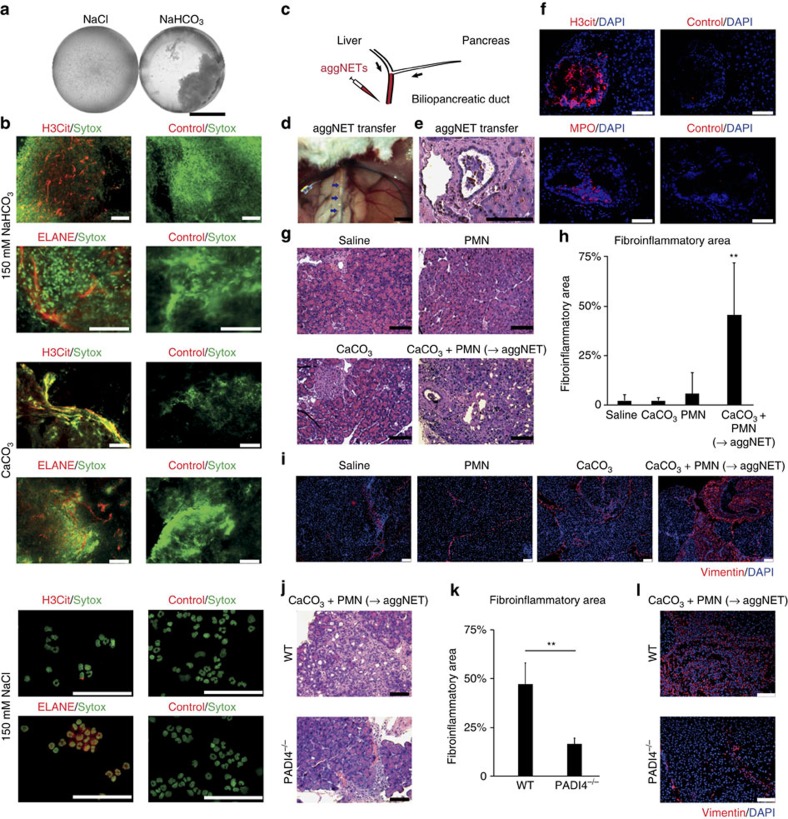
Intraductal formation of carbonate-induced aggNETs causes segmental pancreatic atrophy. (**a**–**c**) Neutrophil-rich peritonitis was induced in mice by means of intraperitoneal thioglycolate injection. After 18 h, 2 ml of 150 mM sodium bicarbonate, calcium carbonate crystals (20 mg) or 2 ml of saline control were injected into the peritoneal cavity. Thirty minutes later, a peritoneal HBSS lavage was aspirated, aggregates were collected and processed for consecutive immunocytochemistry; (**a**) note that aggNETs visible to the naked eye could only be aspirated after injection of NaHCO_3_ but not in saline control (**b**). Carbonate-induced aggNETs formed *in vivo* were immunopositive for citrullinated histone H3 and neutrophil elastase (ELANE) (three independent experiments of a total of *n*=6 per group). (**c**) Model of the aggNET transfer technique. We carefully transferred calcium carbonate crystals (0.5 mg) and thioglycolate-induced neutrophils (10^6^ cells) in 10 μl each to the biliopancreatic duct to form aggNETs *in situ*, as well as saline or single-component controls. The biliary duct was ligated with a suture close to the liver hilus. (**d**) Intraoperative situs post aggNET transfer. The biliopancreatic duct filled with aggNETs and the stomach are marked by arrows and asterisks, respectively (bar, 10 mm). (**e**) Pancreatic duct filled with an aggregate after *in situ* aggNET formation (bar, 100 μm). (**f**) Immunohistochemical detection of citrullinated histone and myeloperoxidase in intraductal aggNETs 4 h after aggNET transfer. (**g**) Haematoxylin and eosin staining and (**i**) vimentin immunohistochemistry revealed the marked segmental fibroinflammatory area and mesenchymal cell expansion 6 days after aggNET transfer. (**h**) The sectional area of the pancreas affected by fibroinflammatory remodelling 6 days after aggNET transfer was calculated for each experimental group (two independent experiments, *n*=6 per group, mean+s.e.m., **P<0.01 one-way analysis of variance/*post hoc* Tukey honest significant difference analysis). (**j**–**l**) Crystals and neutrophil preparations of PADI4-deficient and wild-type mice were placed in biliopancreatic ducts of the respective recipients of the same genotype (two independent experiments, *n*=9 per group, **P<0.01 Student's *t*-test). In both experimental groups, fibroinflammatory remodelling was evident (**j**), yet the affected fibroinflammatory area was markedly reduced in PADI4-deficient mice (mean+s.e.m.) (**k**). Mesenchymal expansion as assessed by vimentin immunohistochemistry was attenuated as compared with wild-type controls (**l**). Black scale bars, 200 μm; white scale bars, 50 μm, unless stated otherwise.

To further test our hypothesis of aggNET-mediated ductal occlusion and its PADI4 dependency, we next developed a system of direct aggNET formation *in situ*. In a first step, we tested both bicarbonate and CaCO_3_ as inducers of aggNET formation *in vivo.* Injection of either crystals or sodium bicarbonate into established thioglycolate-induced peritonitis induced the formation of visible aggregates (Fig. 5a,b). These aggregates displayed both ELANE and H3cit positivity (Fig. 5b). In contrast, control saline injection did not induce visible aggregate formation in peritoneal lavages and only few single H3cit-positive cells were detected by microscopy. To now directly assess the pathological consequences of aggNET formation *in situ*, we injected neutrophils and carbonate crystals into the common biliopancreatic duct (Fig. 5c,d): after injection of the aggNET inducer (CaCO_3_) and neutrophils (PMN), we noted the formation of aggNETs *in situ* (Fig. 5d,e), as MPO- and H3cit-positive intraductal aggregates (Fig. 5f). AggNET formation *in situ* caused acute pancreatitis followed by fibroinflammatory remodelling as assessed by histology (Fig. 5g). The percentage of the pancreatic area affected by fibroinflammatory remodelling was significantly increased after aggNET transfer as compared with single-component injection or saline control (Fig. 5h). Fibroinflammatory remodelling was further analysed by vimentin immunohistochemistry displaying the most pronounced mesenchymal expansion after aggNET transfer (Fig. 5i). In additional experiments, we assessed the functional contribution of PADI4 to this model. Importantly, when *PADI4*-deficient mice were used, fibroinflammatory remodelling was significantly reduced as compared with wild-type controls, as assessed by histology and quantitative morphometry (Fig. 5j, k). In addition, vimentin immunohistochemistry demonstrated that transfer of wild-type aggNETs led to a marked expansion of mesenchymal cells reflecting fibroinflammatory remodelling. In contrast, mesenchymal expansion was markedly attenuated in the setting of *PADI4* deficiency (Fig. 5l). This demonstrates the specific involvement of PADI4 in a second, independent and newly developed model of pancreatitis driven by inflammatory ductal occlusion.

**Figure 6:**
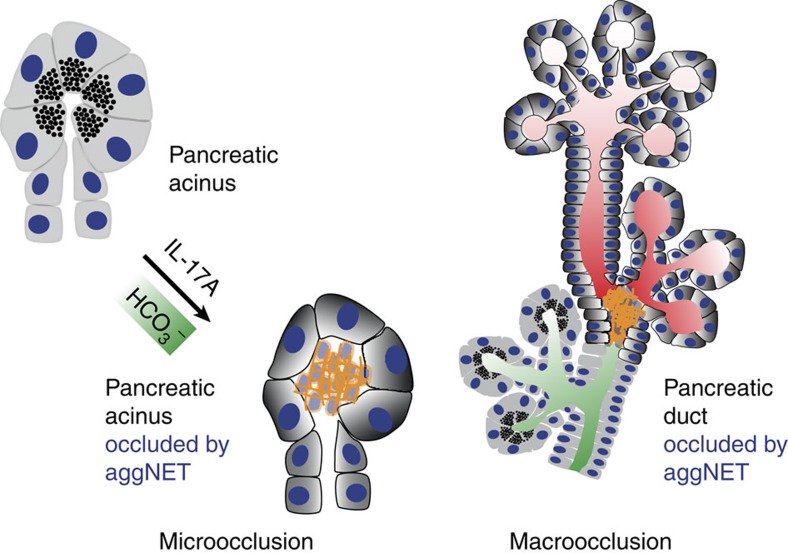
Model of neutrophil-mediated ductal occlusion. Model of intrapancreatic intraductal neutrophil accumulation in response to IL-17A and its downstream targets followed by increased intraductal chromatin extrusion and aggregation in response to HCO_3_^−^ leading to microocclusion on the acinar level (left) or macroocclusion on the lobular ductal level (right).

**Figure 7:**
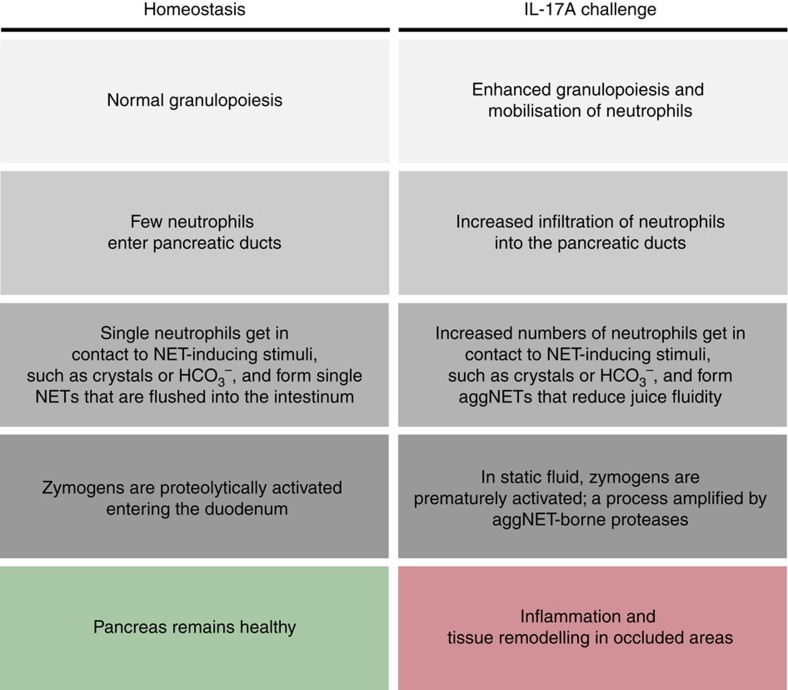
Hypothesis chart. Danger signals, exemplified by IL-17A challenge, instigate enforced granulopoesis, neutrophil mobilization and increased chemoattraction to the pancreas. When neutrophils encounter stimuli in the pancreatic juice such as elevated bicarbonate levels or CaCO_3_ precipitations, they form aggNETs. The large chromatin tangles of the latter reduce the fluidity of the pancreatic juice and consequently hamper secretory flow and lead to focal occlusion of the ductal tree. In occluded areas, digestive zymogens undergo premature activation. AggNET-borne serine proteases in static fluid may amplify this process. Dependent acini are destroyed, inflammation is perpetuated and finally tissue remodelling ensues.

Taken together, we describe intraductal aggNETs as an important element of pancreatic inflammation (Figs 6 and 7). Various signals^49^, including IL-17A-induced factors, instigate increased neutrophil chemoattraction to the pancreatic duct. Once transmigrated to the pancreatic duct, neutrophils encounter stimuli in the pancreatic juice such as increased bicarbonate concentrations or precipitations of CaCO_3._ In high cellular density, this results in intraductal aggNET formation. Large chromatin tangles reduce the fluidity of the pancreatic juice and consequently hamper secretory flow. This leads to focal occlusion of the pancreatic ductal tree, destruction of dependent acini and perpetuation of inflammation. In addition, bicarbonate-induced aggNETs contain serine proteases, prone to initiate pancreatic autodigestion by premature zymogen activation in static fluids^33^,^50^,^51^,^52^. We speculate that aggNETs may furthermore provide a nidus of pancreatic stone formation^53^. Deficiency of neutrophil-mediated arginine citrullination and chromatin decondensation as in PADI4^−/−^ mice protects from pancreatitis in two distinct experimental models (IL-17A delivery, aggNET transfer), whereas *PADI4* deficiency cannot disrupt pancreatitis mediated by acinar cell toxicity (caerulein). The principle of ductal occlusion by aggNETs might also be applicable to various inflammatory conditions involving secretory ducts. Furthermore, our study highlights the role of bicarbonate and CO_2_ in the extrusion of decondensed neutrophil chromatin both *in vitro* and *in vivo*. Future studies on the molecular mechanisms of NET formation need to carefully consider the role of the bicarbonate–CO_2_ rheostat in their experimental systems.

## Methods

### Mice

The mouse lines used have all been described previously: Villin-*Cre*^54^, IL-17A^ind^ (ref. 55), Rag1^−/−^, (JAX, Bar Harbor, ME, USA), Tnfr1+2^−/−^ (JAX), Il6^−/−^ (JAX), Il1r1^−/−^ (kindly provided by E. von Stebut, University of Mainz, Germany) and PADI4^−/−^ (kindly provided by K. Mowen, Scripps Institute, La Jolla, CA, USA). All mouse lines are on the C57Bl/6 background. Wild-type C57Bl/6J animals were bred locally or bought from Charles River, Sulzfeld, Germany. Both sexes of mice were used throughout the studies. For each individual experiment, age- and sex-matched mice were used. Mice aged 6–14 weeks were used for experimental procedures. All mice were kept under specific pathogen-free conditions at the animal facilities of the Universities of Mainz and Erlangen, respectively. Experimental procedures were approved by the local committees of Rhineland-Palatinate and Middle Franconia, respectively.

### Experimental models of disease

Caerulein-induced pancreatitis was induced by 2 consecutive days of intraperitoneal caerulein injections repeated hourly (nine injections per day, 50 μg kg^−1^ per injection, Sigma-Aldrich), followed by lipopolysaccharide (1 mg kg^−1^, Sigma) intraperitoneally at the end of day 1. Mice were starved overnight before caerulein injections. Blood was drawn from the facial veins after 36 h for serum amylase and lipase activity quantification. Mice were killed 48 h after the first injection for histological analysis of pancreata and lung samples. For aggNET transfer experiments, the protocol was adapted from Laukkarinen *et al*.^56^. Briefly, surgery was performed after skin shaving under a stereomicroscope. Intraoperative anaesthesia was achieved with a ketamine/xylazine cocktail (100 and 10 mg kg^−1^, respectively) supplemented with continuous titrated isoflurane application. The common bile duct was identified in the continuation of the papilla of Vater to the liver hilus and was ligated with a 7-0 prolene suture close to the liver hilus. The papilla of Vater was identified at the duodeno–pancreatic junction on the posterior surface of the duodenum. A 30-G cannula was inserted distally into the common bile duct, well before the entry of the main pancreatic duct. Ten microlitres each of 150 mM saline, cell suspension in saline and/or carbonate crystals in saline (10^8^ cells per ml; CaCO_3_: 50 mg ml^−1^) were placed in the common biliopancreatic duct. The laparotomy was closed in two layers; skin closure was performed with Michel suture clips. Mice were given free access to water and regular chow, and post-operative analgesia was achieved by administration of buprenorphine hydrochloride in an 8-h interval for 72 h. The entire surgery duration did not exceed 20 min, and animal survival exceeded 90%. The atrophic area was calculated based on the sections of both the mesenteric region and the body of the organ. For the detection of exocrine pancreatic insufficiency, faecal pellets were collected under standard lab chow (5% raw fat) diet and after 24 h of a high-fat diet (22% raw fat). PBS was added to faecal pellet homogenates and the supernatant was subjected to biochemical analysis.

### *In vivo* imaging

COLOVIEW high-resolution mouse video endoscopic system (Karl Storz, Tuttlingen, Germany) was used for mouse colonoscopy^57^. Bioluminescent luminol imaging for the detection of active MPO was performed injecting 200 mg kg^−1^ luminol sodium salt (Sigma, Germany) intraperitoneally in a volume of 100 μl PBS. The mice were killed by isoflurane and imaged in the bioluminescence camera (IVIS system, PerkinElmer, USA) 10 min after injection, with 5 min of luminescent exposure, as previously described. Specificity of MPO activity of this assay was previously shown^58^.

### Generation and application of the IL-17A expression vector

The *in vivo* expression construct for IL-17A was cloned in a vector system described previously^59^. In brief, the full coding sequence of murine IL-17A including the N-terminal secretion signal was amplified from complementary DNA (cDNA) of *in vitro* generated Th17 cells by reverse transcription PCR and consecutively cloned downstream of a Kozak consensus sequence into a vector system enabling long-term hepatocyte-specific protein overexpression. Plasmid DNA was isolated from Maxi Preps with Qiagen Plasmid Gigakits including endotoxin removal. For *in vivo* gene transfer, 10 μg of IL-17A expression vector or 10 μg empty control vector was injected into the tail vein of recipient mice, respectively.

### Cell isolation procedures

Murine pancreatic cells were isolated from pancreatic tissue using a modified protocol. In brief, pancreatic tissue was mechanically dissected and thoroughly washed in PBS. Tissue was incubated in 5 ml digestion solution containing 0.05 g of collagenase D (Roche Diagnostics, Mannheim, Germany), 0.05 g of DNase-I (Sigma-Aldrich, Munich, Germany) and 0.3 g of dispase II (Roche Diagnostics) in Hank's balanced salt solution (HBSS) for 20 min at 37 °C at slow rotation. Digestion and intermittent washing in HBSS were repeated three times. Digested tissue was passed through a 100-μm cell strainer. Pancreatic acini were microscopically identified and directly stimulated for 10 h as indicated. Viability of cells was checked via a trypan exclusion test. For mesenchymal cell isolation, non-adherent cells and dead acinar cells were removed after overnight culture. Remaining cells grown to confluent monolayers showed fibroblastoid morphology. Culture media contained penicillin and streptomycin. Passages two to six were used for *in vitro* stimulation experiments. Recombinant murine IL-17A (100 ng ml^−1^) and IL-1β (10 ng ml^−1^) were purchased from Immunotools, Friesoythe, Germany. Infiltrating leukocytes from pancreatic tissue were isolated using an intestinal lamina propria isolation kit following manufacturer's instructions (Miltenyi, Bergisch Gladbach, Germany). Murine peritoneal neutrophils were isolated 18 h after intraperitoneal thioglycolate injection and were consecutively cultured in calcium and magnesium containing HBSS medium supplemented with 1% bovine serum albumin (BSA) for up to 4 h. *In vivo* peritoneal aggNET formation was achieved by injection of 150 mM NaHCO_3_ and 20 mg CaCO_3_, respectively, 18 h after initial thioglycolate injection. Peritoneal aggNETs were aspirated by HBSS lavage and consecutively hand-picked from wells for immunocytochemistry. Human peripheral blood neutrophils were isolated from healthy donors and separated using either PanColl (PanBiotech, Germany) or Lymphoflot (Bio-Rad) density gradient centrifugation. In case of PanColl-based isolation, granulocytes were enriched from the erythrocyte pellet by consequent dextrane sedimentation (60 min, 1%, Carl Roth, Germany). In case of Lymphoflot-based isolation, the granulocyte-containing layer on top of the erythrocyte-containing layer was used. Hypotonic lysis removed remaining erythrocytes. The purity of neutrophil isolations was routinely above 90%. In some experiments, cells were preincubated with the PAD4 inhibitor Cl-amidine (0.2–1 mM, Calbiochem) or dimethylsulphoxide control for 30 min before stimulation.

### Cytokine/enzyme quantification and clinical chemistry

Intestinal and colonic sections (equal in size) were cultured for 24 h in RPMI 1640+penicillin/streptomycin (Biochrom, Berlin, Germany). Serum IL-17A and IL-6 levels were quantified using an enzyme-linked immunosorbent assay kit (eBioscience, San Diego, CA) as per the manufacturer's recommended protocol. Faecal triglycerides, amylase and lipase activities were measured using an automated photometric method in the central clinical laboratories of the University Clinics of Mainz and Erlangen. Blood glucose levels were measured using the Roche Aviva handheld system (Roche, Penzberg, Germany). For tryptic activity measurements, pancreata were mechanically homogenized on ice in a buffer containing 5 mM MES (pH 6.5), 1 mM MgSO_4_ and 250 mM sucrose. Protein concentration was determined by Bradford assay. An aliquot of the homogenate was added to the assay buffer containing 50 mM Tris-HCl (pH 8.0), 150 mM NaCl, 1 mM CaCl_2_ and 0.1 mg ml^−1^ BSA. The reaction was started by adding a specific substrate, Boc-Gln-Ala-Arg-MCA, which is converted to a fluorescent product that emits fluorescence at 440 nm upon excitation at 380 nm. Fluorescence was determined at 37 °C. The increase in fluorescence was linear during the observation period (5 min). For the detection of tryptic activity in pancreatic juice samples, the specific substrate was added directly to the pure juice sample and fluorescence was quantified. Clinical blood–gas analysis of pancreatic juice was performed after thawing before performing the neutrophil stimulation experiments using a point-of-care analyser (Radiometer, Germany). DNA was quantified fluorimetrically in a Tecan M200 fluorometer using Sytox Green (Molecular Probes, 2.5 μM); in some experiments, CO_2_ control was achieved during the analysis by use of a gas-control module (Tecan M200Pro, as indicated).

### Real-time quantitative PCR

Tissue RNA was isolated by directly freezing tissue samples in liquid nitrogen in lysis buffer of the peqGOLD Total RNA Kit. Cell culture RNA was isolated using the Qiagen MicroKit (Qiagen, Hilden, Germany). RNA quantification was performed using Nanodrop technology (Thermo Scientific, Wilmington, DE). Reverse transcription into cDNA was performed using the Bio-Rad iScript cDNA synthesis Kit (Bio-Rad Laboratories, Munich, Germany). As a quality control, reverse transcription PCR for *Bact* was performed and only samples with a positive PCR product after 30 cycles were used for subsequent quantitative PCR studies. Quantitative PCR was performed using QuantiTect Primer Assays for *Bact*, *Ctgf*, *Cxcl1*, *Cxcl5*, *Hprt*, *Il1b*, *Il6*, *Mmp2*, *Mmp9*, *Nfkbiz*, *Tgfb*, *Tnfa*, *Padi4*, *Pdgfa*, *Pdgfb* and *Timp1* (Qiagen, Hilden, Germany), and QuantiTect SYBR Green RT-PCR Kit (Qiagen) on the Roche LightCycler system (Roche, Penzberg, Germany). Expression was calculated relative to the housekeeping gene *Hprt* using the delta–delta threshold cycle (ΔΔCt) algorithm. Fold difference to control treated animals or unstimulated control, respectively, was calculated as a ratio to the respective control mean.

### *In vivo* circulatory neutrophil depletion

Circulatory neutrophil depletion was performed using a neutrophil-specific anti-Ly6G antibody (clone 1A8, BioXCell, USA). The antibody was injected every other day at a dose of 10 mg kg^−1^. Isotype control antibody (clone 2A3, BioXCell) was injected equally into control animals.

### Immunohistochemistry and flow cytometry

Histochemical staining was performed on paraffin-embedded slides with classical haematoxylin eosin or Masson trichrome staining procedure. Immunofluorescence of cryosections or paraffin-embedded slides was performed as described below and recorded on either a confocal laser scanning microscope or a standard fluorescence microscope (Leica, Germany) using overnight hybridization with primary antibodies specific for α-smooth muscle actin (Abcam, Cambridge, UK, 1:500), cleaved Caspase 3 (Cell Signaling, NEB, 1:300), Cramp (Innovagen, Lund, Sweden, 1:200), neutrophil elastase (Abcam, 1:200), EpCAM (BioLegend, 1:100), F4/80 (eBioscience, 1:1,000), citrullinated histone H3 (Abcam, 1:200), human IL-17A (R&D Systems, Wiesbaden, Germany, 1:100), mouse IL-17A (Santa Cruz, Heidelberg, Germany, 1:500) and MPO (Abcam, 1:200). Detection was performed using either biotinylated secondary antibodies (goat anti-rabbit or anti-rat, Abcam, 1:1,000) and TSA Fluorescein/Cy3 kits (PerkinElmer, Waltham, MA, USA) or directly labelled Alexa 488 or Alexa 555-conjugated goat anti-rat antibodies (Abcam, 1:200–1:1,000). Before examination, the nuclei were counterstained with Hoechst 33342, propidium iodide or Sytox Green (Invitrogen Molecular Probes, Karlsruhe, Germany; BD, Heidelberg, Germany). TUNEL for the *in situ* detection of cell death was performed using the Roche *in situ* cell death detection kit according to the manufacturer's protocol (Roche, Mannheim, Germany). NET quantification was performed using mean fluorescence intensity and area analysis functions of Adobe Photoshop CS5. Flow cytometry was performed on a BD Fortessa instrument after surface staining of CD11b (1:500), Ly6G (1:500), CD4 (1:500), CD8 (1:500) and B220 (1:500) coupled with different standard fluorophores FITC, PE, PE-Cy7, APC and PerCP-Cy5.5 (BioLegend). Cells were gated on live cells and cell death exclusion was performed using PI (1:100) or 7AAD (1:100) staining before flow cytometry. Recording was performed using FACS DIVA software, further analysis was performed using FlowJo 7.6.5 software.

### Live-cell imaging

Freshly isolated human peripheral blood granulocytes (10^6^) in a volume of 10 μl were carefully added to the bottom of Nunc Lab-Tek II Chamber Slides (Thermo Scientific, Germany) filled with CO_2_-saturated, pre-warmed 150 mM NaHCO_3_ (5% FCS, 1 μg ml^−1^ propidium iodide (Sigma, Germany), 1 μg ml^−1^ Hoechst 33342 (Molecular Probes, Netherlands)). Cells were incubated at 37 °C and monitored in parallel via fluorescence microscopy in a time-lapse manner for up to 60 min at different magnifications (× 20 and × 60). Photos were processed in Adobe Photoshop CS5. For live-cell imaging of intracellular pH (pHrodo Red AM, Molecular Probes), cells were cultured at 37 °C/5% CO_2_ in an incubation chamber and subjected to isotonic HBSS supplemented with 50 mM NaHCO_3_ and 1% BSA.

### Intracellular pH quantification

For the flow cytometric measurement of intracellular pH, human granulocytes were incubated with the pH indicator SNARF-1 (Life Technologies, Germany) and the fluorescent emissions at various concentrations of NaHCO_3_ were recorded with a Gallios Flow Cytometer (Beckman Coulter, USA). The shift from 575 to 660 nm emission was considered as an indicator of pH and was evaluated with the Beckman Coulter analysis software Kaluza 1.3.

### Ratiometric calcium quantification

Freshly isolated neutrophils were adjusted to a final concentration of 10^7^ cells per ml and loaded with Fluo-3 AM (Life Technologies) and Fura-Red AM (Life Technologies) at a final concentration of 3 and 6 μM, respectively. Cells were incubated for 30 min at room temperature. Cells were washed twice with HBSS and adjusted to a final concentration of 5 × 10^6^ cells per ml. Subsequently, cells were subjected to flow cytometric analysis. The samples were measured for 30 s, upon which an equal amount of HBSS or isotonic HBSS containing 75 mM bicarbonate were added. The sample was analysed for a period of 15 min. For the measurement, a modified Gallios flow cytometer was used (Beckmann Coulter). Subsequent analysis was assisted by the software Kaluza 1.3 (Beckmann Coulter). Events of each minute of measurement were integrated and the ratio of FL1 to FL3 was determined as being indicative for intracellular calcium levels. The ratiometric calcium concentration was normalized to the baseline ratio before stimulation.

### Calculation of affected area

Tissue sections stained with haematoxylin and eosin were used for a blinded morphometric analysis calculating the area affected by fibroinflammatory remodelling relative to the total sectional area. Calculation of the affected area was assisted by Image J.

### Human samples

Paraffin-embedded tissue (5 samples of chronic pancreatitis and 8 samples of malignancy-related inflammation), pancreatic juice samples (*n*=20; 10 patients with pancreatic malignancy, 10 patients with benign (chronic) pancreatitis, including 5 patients with hereditary pancreatitis) were collected during endoscopic retrograde pancreaticography and fresh punctates of dilated pancreatic ducts and pseudocysts during endosonography (*n*=3, benign pancreatitis). Pancreatic juice was snap-frozen after isolation and stored at −80 °C until analysis. All procedures were performed for medical reasons during routine clinical practice after informed consent and ethical review of the local authorities of Trier, Erlangen and Greifswald.

### Reagents

To ensure isotonicity of bicarbonate-containing HBSS-based buffers, various amounts of a solution of 150 mM NaHCO_3_ in salt-free water (300 mOsm kg^−1^) were added to isotonic HBSS supplemented with 1% BSA and 1 mM CaCl_2_. Salinity of the resulting bicarbonate-containing buffers was checked before use. To avoid issues of stability, the bicarbonate solution was maintained at 4 °C in tightly sealed containers and was freshly prepared every 2 weeks. Bicarbonate-containing HBSS solutions were freshly prepared on each experimental day.

Calcium carbonate crystals (aragonite) were generated according to established protocols^44^. Twenty-five millilitres of 1 M Ca(NO_3_)_2_ × 4 × H_2_O and 250 ml of 0.1 M Na_2_CO_3_ were heated to 70 °C. The Ca-solution was slowly added to the carbonate solution, and the mixture was stirred for 30 min at 70 °C. Crystals were collected by filtration, washed sequentially with water and ethanol and dried at 100 °C. A final concentration of 1–5 mg ml^−1^ was used for cell culture stimulation.

### Statistical analysis

Data were analysed by the unpaired Student's *t*-test using Microsoft Excel (Microsoft, Redmond, WA) or analysis of variance with *post hoc* Tukey honest significant difference tests, as indicated, using SPSS software. Figure preparation was performed using FlowJo 7.6.5, Adobe Creative Suite CS5 and the Microsoft Office Suite 2010.

## Additional information

**How to cite this article:** Leppkes, M. *et al*. Externalized decondensed neutrophil chromatin occludes pancreatic ducts and drives pancreatitis. *Nat. Commun.* 7:10973 doi: 10.1038/ncomms10973 (2016).

## Supplementary Material

Supplementary Information Supplementary Figures 1-9

Supplementary Movie 1 Human neutrophils underwent chromatin decondensation and rapid extrusion, when cultured in human pancreatic juice at 37°C / 5% CO2 (50% isotonic juice, 50% HBSS isotonic medium).

Supplementary Movie 2 Human PMN subjected to 150 mM NaHCO3 supplemented with 5% FCS underwent morphological changes and forcefully released DNA. Viable PMN showed low propidium iodide (red) and high Hoechst 33342 signal (green).

Supplementary Movie 3 Human neutrophils loaded with an intracellular pH dye (pHrodo), which exhibits red fluorescence only at acidic pH, were cultured at 37°C / 5% CO2 in isotonic HBSS supplemented with 50 mM NaHCO3 in the presence of the non-cell permeant DNA probe Sytox Green. A neutrophil already displaying alkalinized cytoplasmic pH executed sudden DNA release, whereas an adjacent cell, which has still retained its neutral pH did not. A similar effect was also observed in other cells of the cultures.

Supplementary Movie 4 Human PMN underwent cellular spreading and disintegration (notable in differential interference contrast [DIC]), chromatin decondensation (visualized by Hoechst 33342 [blue]) and DNA extrusion (visualized by propidium iodide [red]) in response to 150 mM NaHCO3 supplemented with 5% FCS. Externalized DNA of densely packed PMN leads to aggNET formation. All white scale bars are 50μm.

Supplementary Movie 5 Video microscopy of human neutrophils from healthy donors. Neutrophils were allowed to adhere to the surface of an IbidiTreat-coated Ibidi Flow Chamber (Ibidi, Munich, Germany) for 30 minutes at 37°C / 5% CO2 before supplementation with 75 mM NaHCO3 and further incubation for 120 minutes. DNA was detected by Sytox Green. The total time lapse of the movie is 2 minutes compressed to 7 seconds (16x). Please note the tangles of extracellular DNA derived from granulocytes with non-nuclear decondensed morphology, which are attached to the surface and slightly move in the flow. Further note, that threads of free DNA are captured by immobilized DNA aggregates, increasing the size of the latter (n=3 independent experiments; Movie 5 provided as online movie file only).
