# Supplementary material for: Externalized decondensed neutrophil chromatin occludes pancreatic ducts and drives pancreatitis
Source: Nat Commun. 2016 Mar 11;7:10973. doi: 10.1038/ncomms10973 (PMC4793047; doi:10.1038/ncomms10973)
Supplement: Supplementary Information — Supplementary Figures 1-9 [file ncomms10973-s1.pdf]

Supplementary Figure 1

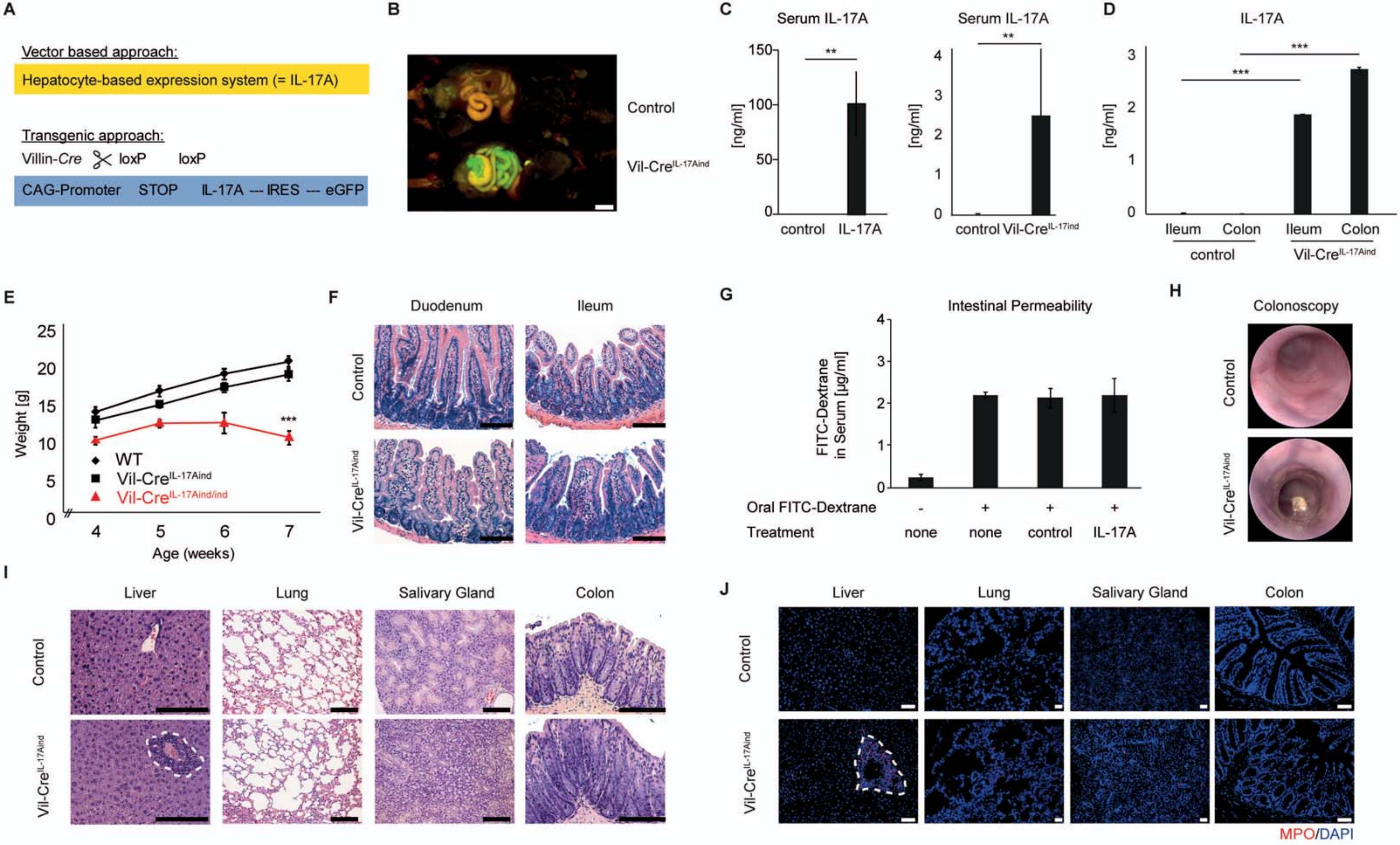

Supplementary Figure 1: Delivery of IL-17A in mice

(A) (upper panel) Expression vector application leads to systemic IL-17A delivery based on albumin promoter directed hepatic gene expression (referred to as IL-17A); (lower panel) genetic construct of Villin Cre – IL-17A^{ind} mice. IL-17A and eGFP are conditionally expressed under control of the Villin promoter in intestinal epithelial cells. **(*B*)** Fluorescence *in vivo* imaging using the Maestro system showing *Cre* mediated intestinal eGFP expression (white scale bar equals 1 cm). ***(C, D)*** After IL-17A delivery, IL-17A levels are robustly elevated in murine sera ***(C)*** and in isolated intestinal organ cultures ***(D)*** (serum and organ culture n=11 and 4 samples per group, respectively). ***(E)*** Weight course showing initial weight gain in all three mouse cohorts. Note that Vil-Cre – IL-17A$^{ind/ind}$ mice failed to gain weight (week 6) and start losing weight from week 7 (n≥6 per group). ***(F)*** Analyses by histopathology of the small intestine of Vil-Cre – IL-17A^{ind} mice and control mice using H&E staining revealed regular crypt-villus architecture in transgenic animals and no signs of increased leukocyte infiltration. ***(G)*** Intestinal permeability was assessed by oral gavage of FITC-dextrane 4kD. No differences in serum levels between untreated, control or IL-17A vector-treated mice was observed (n=4 per group, two independent experiments were performed). ***(H)*** Mouse colonoscopy did not reveal macroscopic signs of colonic inflammation (n= 5 per group, postnatal day 40) ***(I)*** Liver sections reveal a moderate leukocyte infiltration around the Glisson triad area, containing bile duct, liver artery and portal vein (highlighted by white line) (n=8 per group). Sections of lungs, colons and salivary glands do not show tissue destruction or increased leukocyte infiltration. **(J)** MPO immunohistochemistry shows a marked infiltration of MPO^{+} cells in the liver surrounding a central lumen (highlighted by a white line), whereas lungs and salivary glands contain very few infiltrating neutrophils. No granulocyte infiltration was detected in the large intestine (n=8 per group). All black scale bars represent 200 µm, all white bars 50 µm, unless stated otherwise. , ***: $p<0.001$, Student's t-test

Supplementary Figure 2

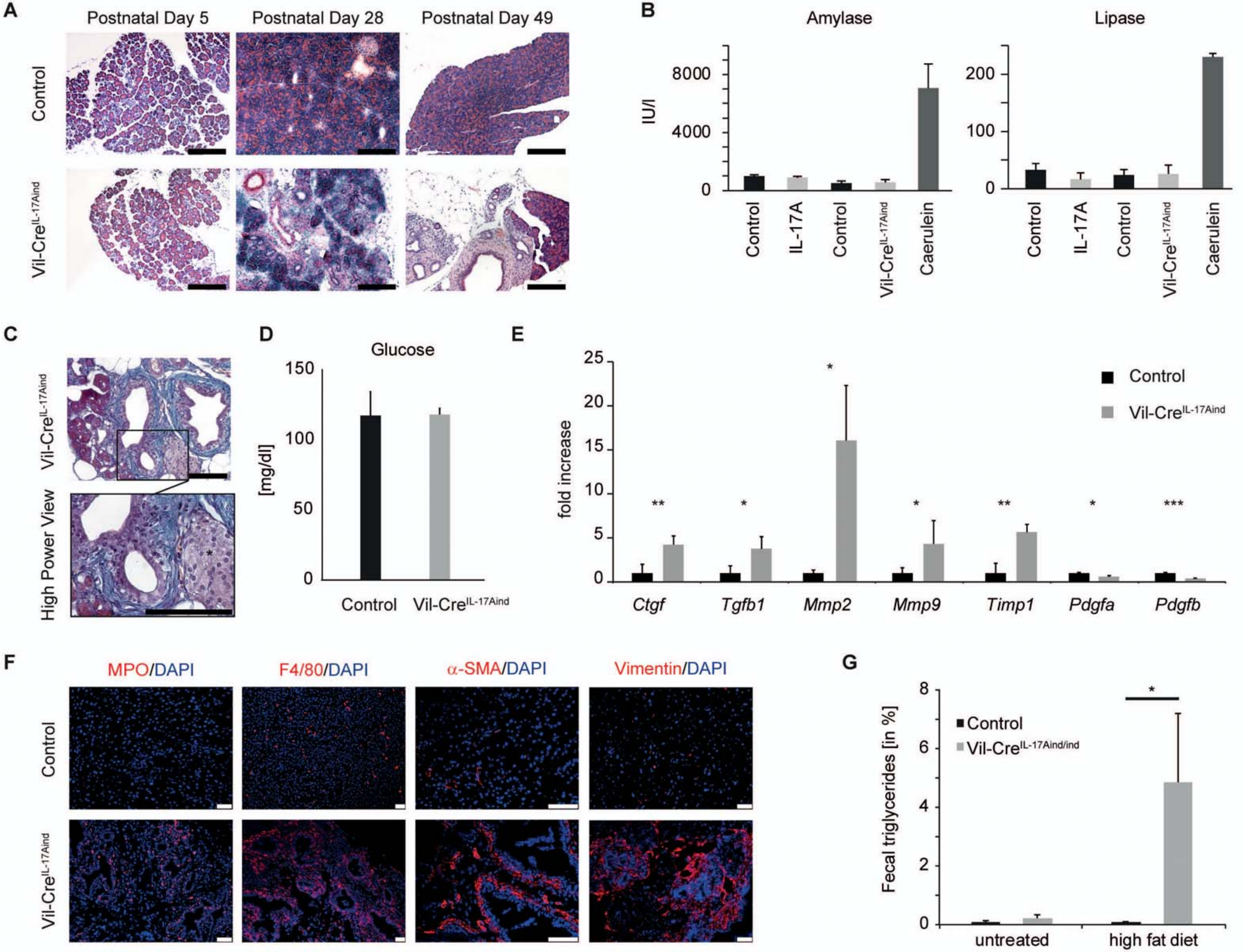

Supplementary Figure 2: Systemic delivery of IL-17A induces infiltration of myeloid cells and tissue fibrosis of the pancreas.

(A) Analysis of various time points reveals regular embryonic development of the pancreas in both experimental groups and infiltration of immune cells followed by progressive tissue remodeling in Vil-Cre – IL-17A^{ind} mice (n≥5 per group and time point). ***(B)*** Despite overt pancreatitis, serum amylase and lipase activities did not increase after IL-17A delivery (vector-based approach: day 7 p.i., transgenic approach: postnatal day 28, n=8 per group, caerulein positive control: day 1 p.i.). ***(C)*** Masson-Goldner trichrome staining shows severe fibrosis, glandular atrophy and pseudotubular structures in pancreas sections of Vil-Cre – IL-17A^{ind} mice, yet intact Langerhans islets (asterisk). ***(D)*** Blood glucose levels of IL-17A transgenics or control mice after 4 hours of fasting showed similar values at postnatal day 36, excluding overt endocrine insufficiency (n= 5 per group). ***(E)*** qPCR analysis of pancreatic tissue (postnatal day 49) of markers for tissue remodelling in wildtype and Vil-Cre – IL-17A^{ind} mice reveals differential expression of several molecules implicated in pancreatic fibrogenesis and tissue remodelling (n=6 per group). ***(F)*** Immunofluorescence shows marked infiltration of MPO^{+} granulocytes and F4/80^{+} macrophages into the pancreata of transgenic mice. The analyses of α-smooth muscle actin (α-SMA) and vimentin expression demonstrates an increase in activated pancreatic mesenchymal cells and tissue fibrosis after IL-17A delivery. ***(G)*** Fecal pellets were collected before and after 24 hours of high fat diet from IL-17A transgenics, that experienced weight loss, and control littermates. Triglycerides were measured in the fecal homogenate. This revealed exocrine pancreatic insufficiency in IL-17A transgenics (n=6 pellets per group of 3 mice per group). All black scale bars represent 200 µm, all white bars 50 µm, unless stated otherwise. (* $p<0.05$, ** $p<0.01$, *** $p<0.001$, Student's t test)

Supplementary Figure 3

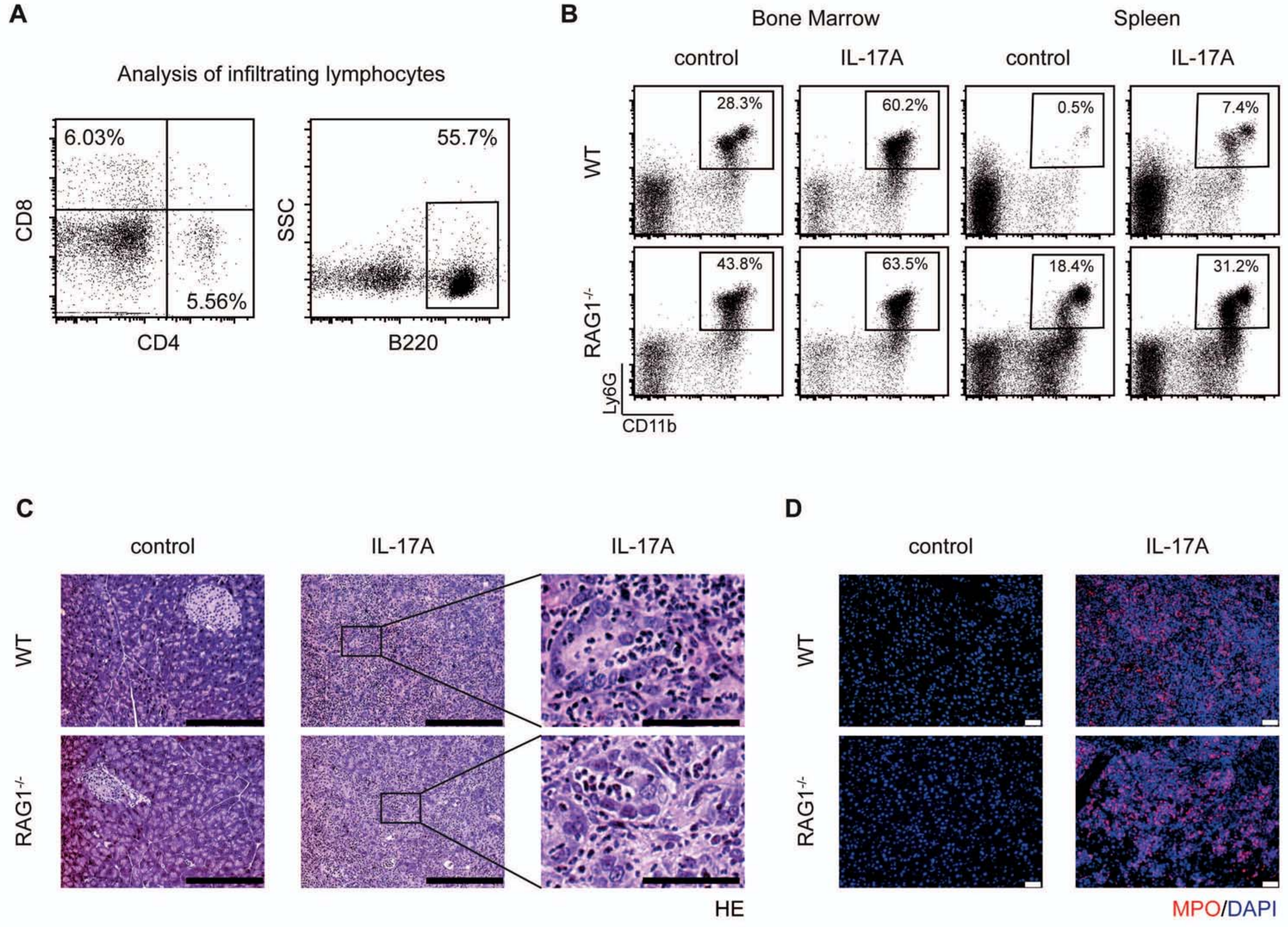

Supplementary Figure 3: The induction of pancreatitis by IL-17A is lymphocyte-independent.

(A) Lymphocytes infiltrating the pancreata after IL-17A delivery were characterized by staining for CD4, CD8 and B220. The pancreata contained a small evenly distributed fraction of CD4 and CD8 positive T cells. The majority of infiltrating lymphocytes expressed B220 on their surfaces. ***(B-D)*** Wildtype and $Rag1^{-/-}$ mice were treated with the IL-17A vector: ***(B)*** Surface staining for CD11b and Ly6G demonstrates enforced granulopoesis in the bone marrow and mobilisation of neutrophils to the spleens independent of the presence of functional B and T cells in $Rag1^{-/-}$ mice. Absence of lymphocytes led to a relative increase in control spleen neutrophils in $Rag1^{-/-}$ mice, which was further enlarged after IL-17A delivery. Note that pancreatitis severity was not altered in Rag1-deficient mice as demonstrated by ***(C)*** H&E staining (scale bars of insets represent 50 µm) and ***(D)*** MPO immunohistochemistry on day 14 after vector injection. Granulocyte infiltration and aggregation occurs in the absence of functional lymphocytes (n≥11 per group). All black scale bars represent 200 µm, all white bars 50 µm, unless stated otherwise.

Supplementary Figure 4

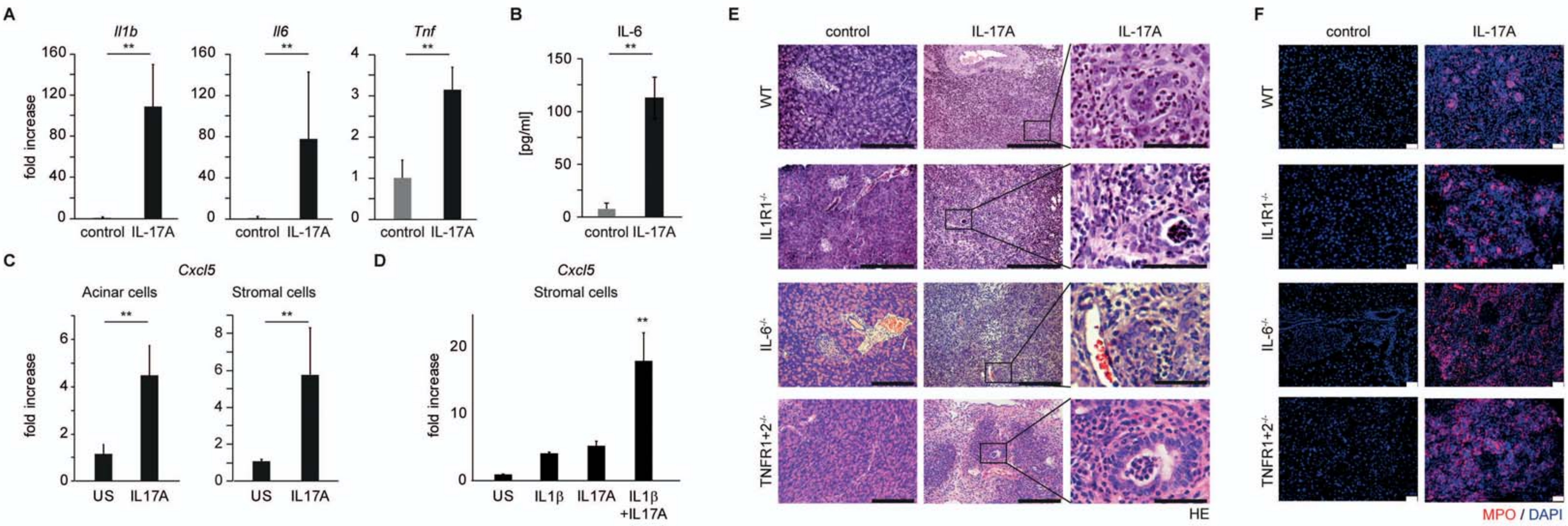

Supplementary Figure 4: IL-17A-induced pancreatitis requires neither IL-1, IL-6 nor TNFα.

(A) qPCR of vector-treated pancreatic tissue reveals a markedly increased expression of *Il1b*, *Il6* and *Tnf* mRNA after IL-17A delivery (day 10 p.i., n=8 per group, ** p<0.01, Student's t test).***(B)*** Mice show elevated serum levels of IL-6 in response to IL-17A (day 10 p.i., n=10 per group derived from at least two independent experiments). ***(C)*** Recombinant murine IL-17A (100 ng/ml) induces expression of *Cxcl5* in pancreatic acinar and stromal cells. RNA was isolated 20 hours after stimulation with the cytokine (1/5 independent experiments is shown, ** p<0.01, Student's t test). ***(D)*** Stimulation with recombinant IL-1β (10 ng/ml) and IL-17A (100 ng/ml) synergistically induces the expression of *Cxcl5* mRNA in pancreatic mesenchymal cells. RNA was isolated 20 hours after cytokine stimulation (1/3 independent experiments is shown, ** p<0.01, one-way ANOVA / postHoc Tukey HSD) ***(E)*** H&E staining of WT control, IL1R1$^{-/-}$, IL6$^{-/-}$ (all day 10 p.i.) and TNFR1+2$^{-/-}$ (day 14 p.i.) mice after IL-17A vector injection versus mock treatment reveals a similar extent of leukocyte infiltration and exocrine pancreatic damage in all IL-17A-treated groups (scale bars of insets represent 50 µm). ***(F)*** MPO immunohistochemistry of pancreatic sections of WT controls, IL1R1$^{-/-}$, IL6$^{-/-}$, TNFR1+2$^{-/-}$ mice after IL-17A vector injection shows marked granulocyte infiltration in all treated groups. Mock treated animals did not show neutrophil infiltration (n≥6 per group). All black scale bars represent 200 µm, all white bars 50 µm, unless stated otherwise.

Supplementary Figure 5

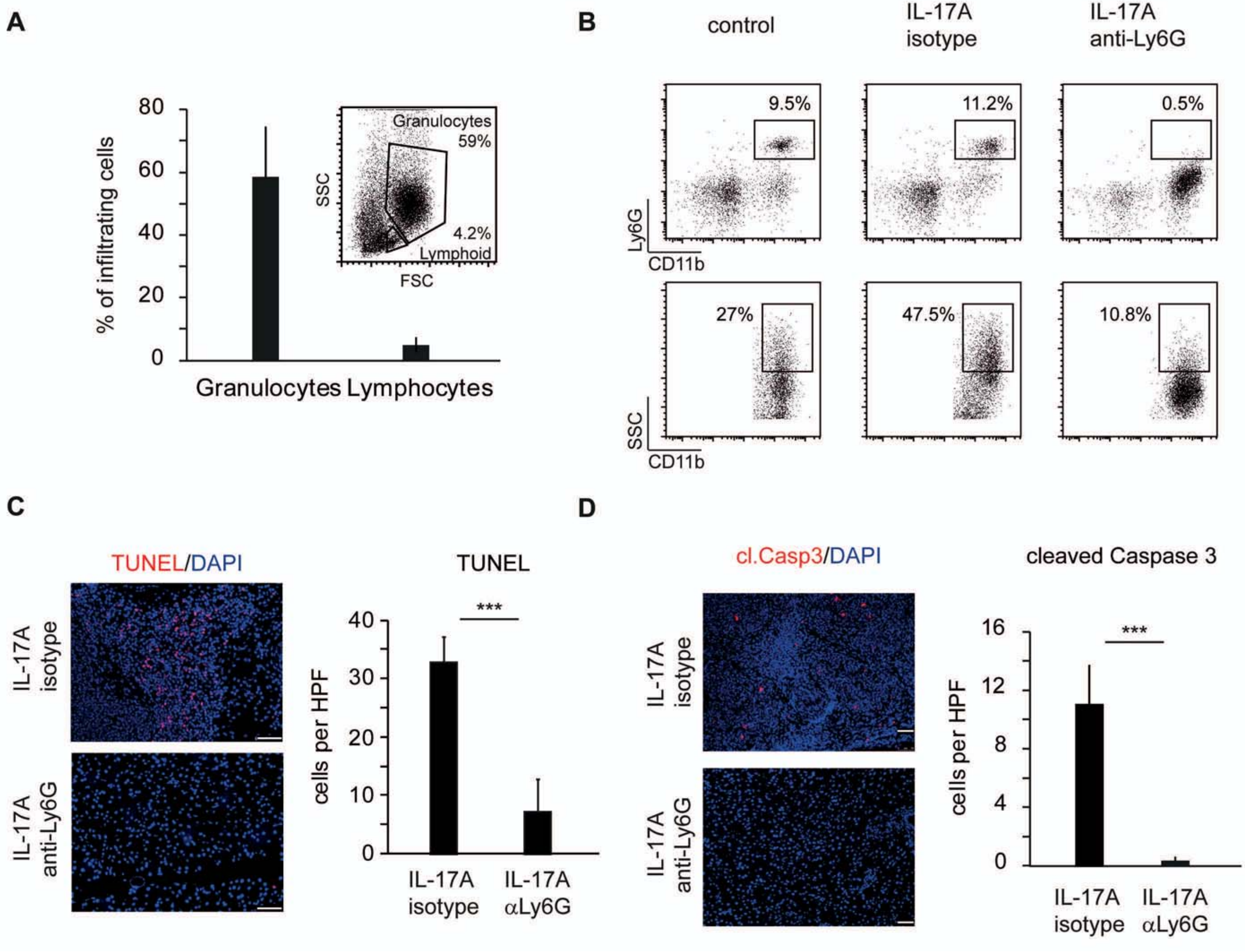

Supplementary Figure 5: The induction of pancreatitis by IL-17A depends on neutrophils.

(A) At day 13 after IL-17A delivery, pancreatic tissue-infiltrating leukocytes were isolated and subjected to analyses by flow cytometry. FSC/SSC scatter plot (inset) shows the gating strategy for $FSC^{hi}SSC^{hi}$ granulocytes and $FSC^{med}SSC^{lo}$ lymphocytes. Note the high and low percentages of infiltrating granulocytes and lymphocytes, respectively. ***(B)*** Flow cytometry of peripheral blood shows increased amounts of circulating $CD11b^{+}SSC^{hi}$ and $CD11b^{+}Ly6G^{+}$ granulocytes after IL-17A delivery. After successful anti-Ly6G treatment, demonstrated by the lack $CD11b^{+}Ly6G^{+}$ stained cells **(upper panel)**, the amount of circulating $CD11b^{+}SSC^{hi}$ granulocytes was strongly reduced even in the setting of IL-17A-induced neutrophilia **(lower panel)**. ***(C, D)*** The quantification by immunofluorescence of $TUNEL^{+}$ cells shows the strongly reduced numbers of cells undergoing cell death in the pancreata of IL-17A expressing mice after neutrophil depletion. The isotype antibody was without protective effect. (analysis of 5 high power fields per slide, $p<0.001$) ***(D)*** The quantification by immunofluorescence of cells that contained cleaved Caspase 3 shows the strongly reduced numbers of apoptizing cells in the pancreata of IL-17A expressing mice after neutrophil depletion. The isotype antibody was without protective effect. (n=4 per group, analysis of 5 high power fields per slide, $p<0.001$). All black scale bars represent 200 µm, all white bars 50 µm, unless stated otherwise.

Supplementary Figure 6

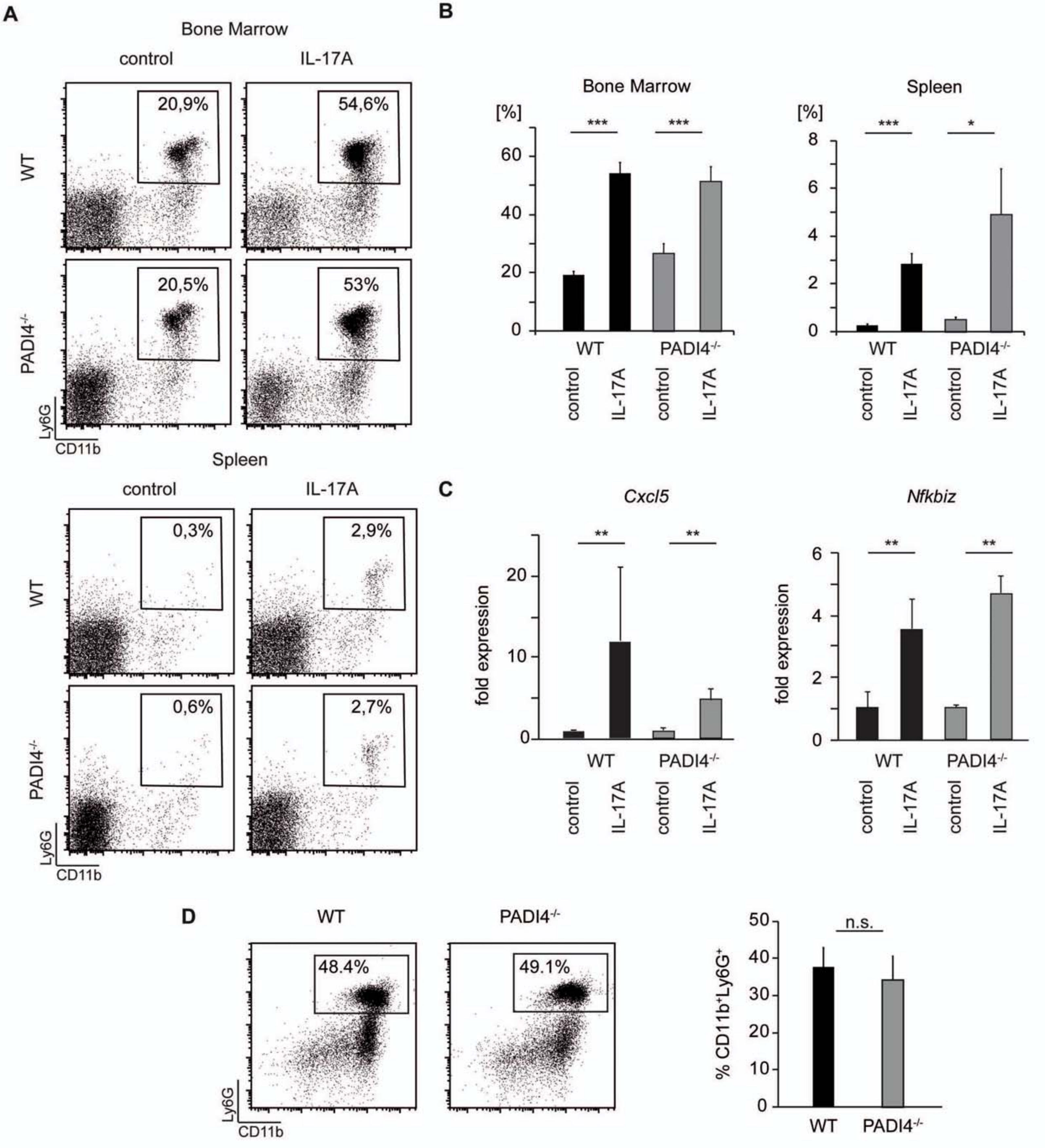

Supplementary Figure 6: PADI4 is required for IL-17A-induced pancreatitis.

(A-C) Wildtype and PADI4$^{-/-}$ mice were treated with the IL-17A vector or mock control. **(A/B)** Analyses by flow cytometry of bone marrow and spleen cells and ***(B)*** respective statistical analysis revealed that IL-17A similarly enforces granulopoesis and neutrophil mobilisation in wild-type and in PADI4 $^{-/-}$ mice. (* $p<0.05$; *** $p<0.01$; Student’s t-test) ***(C)*** mRNA expression analyses of pancreatic tissue showed an increase of both *Cxcl5* and *Nfkbiz* expression after IL-17A delivery. Both wild-type and PADI4-deficient mice showed similar results, when compared to mock treated controls (n=8 per group, ** $p<0.01$ Student’s t-test) ***(D)*** The intrinsic capacity of PADI4-deficient neutrophils to be recruited to the periphery was analysed by means of thioglycolate-induced peritonitis, which showed an equal fraction of infiltrating CD11b^{+}Ly6G^{+} neutrophils 18 hours after injection (>3 independent experiments; n≥5 per group).

Supplementary Figure 7

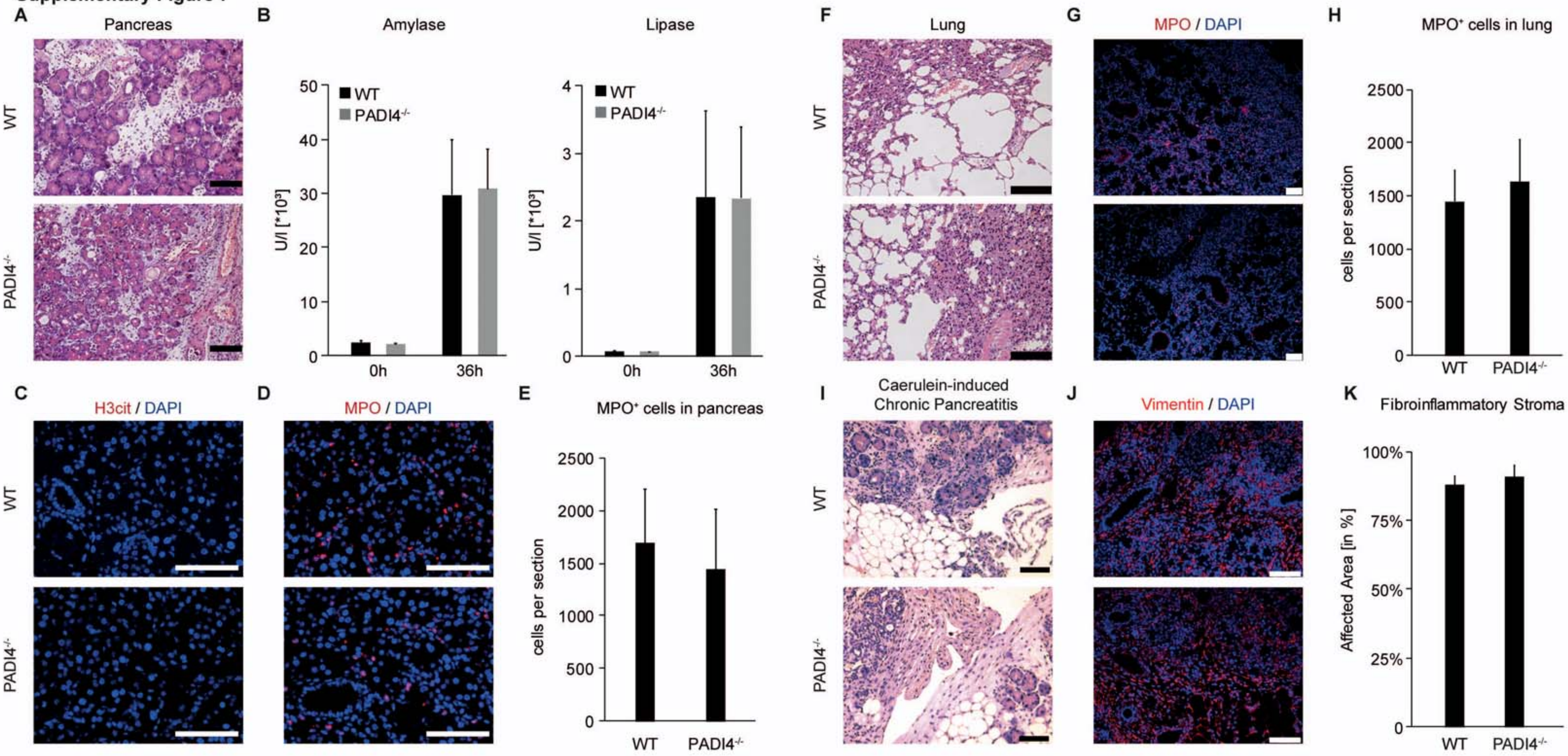

Supplementary Figure 7: PADI4-deficiency does not alter susceptibility to caerulein-induced pancreatitis

In a two-day protocol of caerulein-induced pancreatitis, no difference in susceptibility to disease was noted between wild-type and PADI4-deficient mice (3 independent experiments, n=15 per group). ***(A)*** Pancreatic edema, immune cell infiltration and acinar necrosis could be noted in both wild-type and PADI4-deficient mice in a comparable manner in H&E staining (48 hours after the first injection of caerulein). ***(B)*** Serum amylase and lipase activities increased in PADI4-deficient mice to a similar extent as compared to wild-type mice, 36 h after the first caerulein injection. ***(C)*** In caerulein-induced acute pancreatitis, H3cit-positive intraductal aggregates were observed in neither wild-type nor PADI4-deficient mice. ***(D)*** Caerulein induces a comparable amount of infiltrating neutrophils to the pancreas in both experimental groups. ***(E)*** MPO^+ cells computationally quantified from images as in (D). ***(F)*** H&E staining reveals pronounced tissue damage and a marked neutrophilic infiltration in the lungs after caerulein-challenge in both wild-type and PADI4-deficient mice. ***(G, H)*** Wild-type and PADI4-deficient mice were indistinguishable with regard to lung infiltration of MPO^+ cells by immunohistochemistry ***(G)*** and computational quantification ***(H)***. In a protocol of caerulein-induced chronic pancreatitis, fibroinflammatory remodeling and mesenchymal cell expansion were analysed in both wild-type and PADI4-deficient mice after three weeks (n=6 per group). PADI4-deficient mice were equally susceptible to chronic caerulein-induced pancreatitis as compared to wild-type as assessed by H&E staining analysis ***(I)***, vimentin immunohistochemistry ***(J)*** and calculation of the area affected by fibroinflammatory remodeling ***(K)***. All black scale bars represent 200 µm, all white bars 50 µm, unless stated otherwise.

Supplementary Figure 8

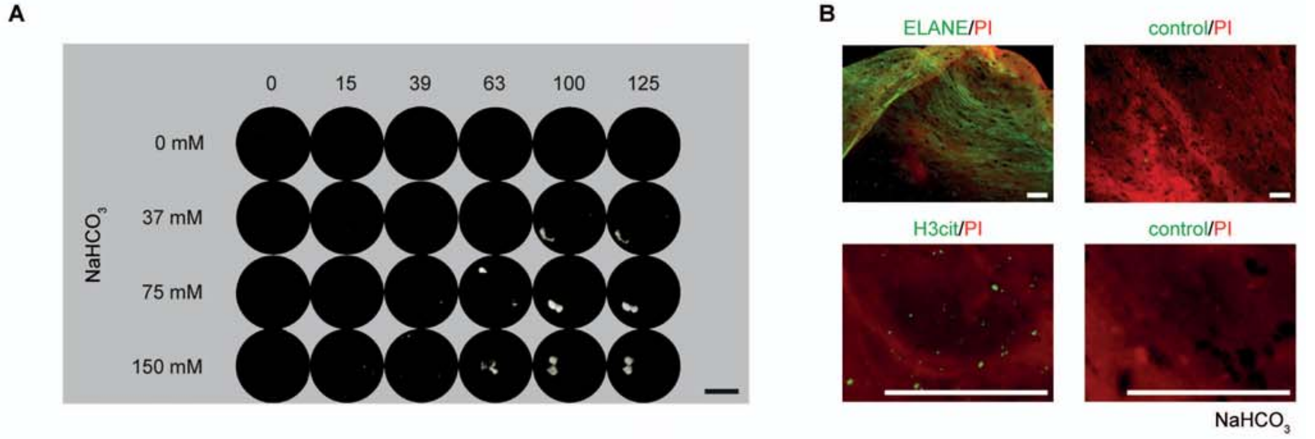

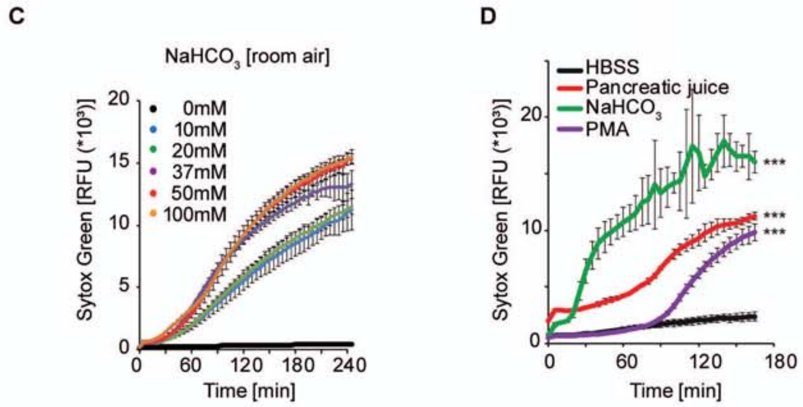

Table 1

Pancreatic juice sample characteristics

pH	7.556		(7.556-8.74)
pCO_2	41.8	mmHg	(7.5-41.8)
HCO_3^-	37	mmol/l	(10.2-91.1)
Hb	0	g/dl	
K^+	29.7	mmol/l	(3.6-29.7)
Na^+	132	mmol/l	(123-208)
Ca^{2+}	1.05	mmol/l	(0.28-1.24)
Cl^-	105	mmol/l	(89-208)
Glucose	50	mg/dl	(1-50)
Lactate	1.5	mmol/l	(0-7.2)
Bilirubin	0	mg/dl	
Osmolality	268	mOsm/kg	(245-417)

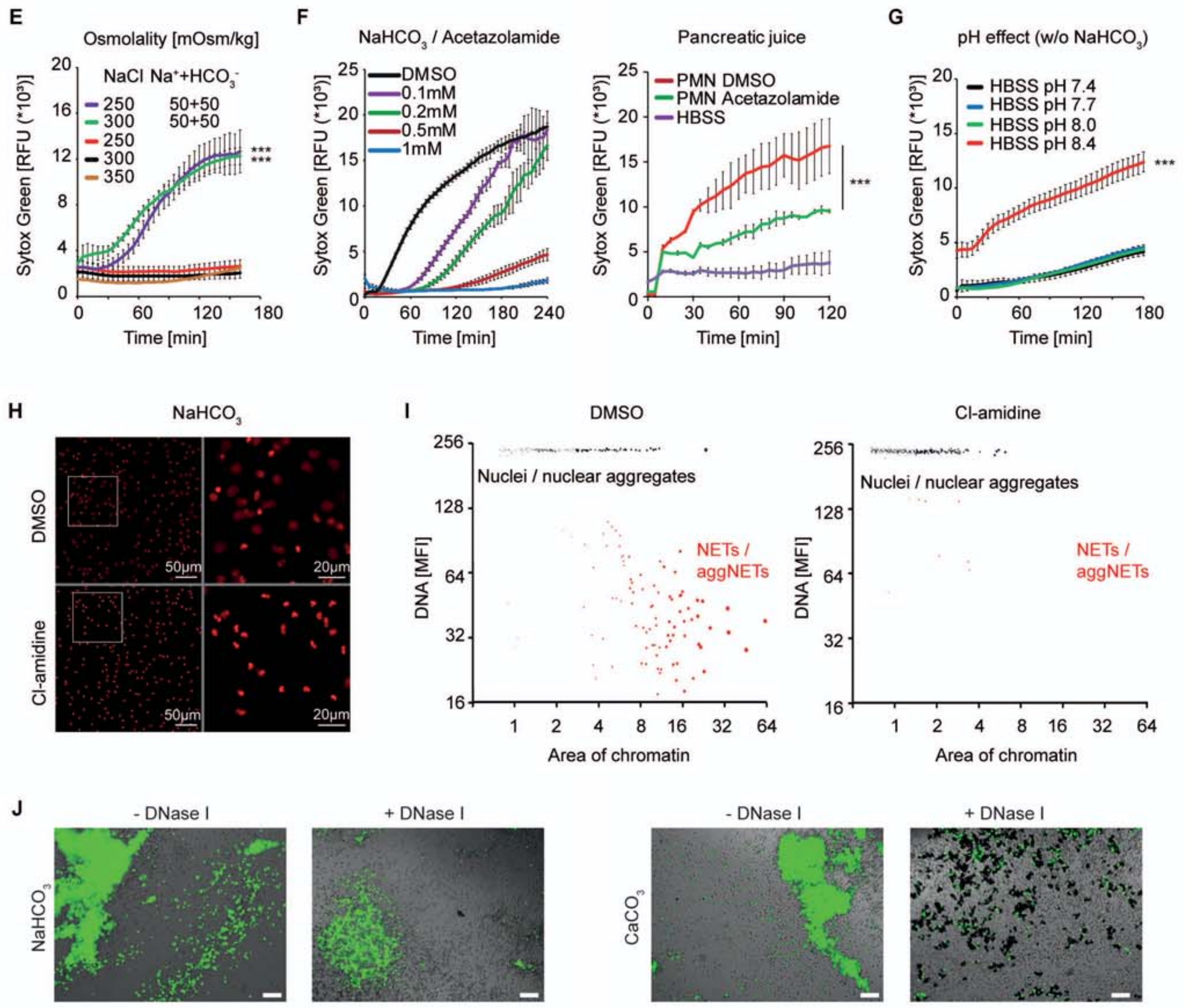

Supplementary Figure 8: Bicarbonate induces chromatin externalisation prone to aggregate.

(A) Freshly isolated neutrophils from healthy donors cultured in isotonic sodium bicarbonate-substituted media showed formation of aggNETs, visible to the naked eye. Bar=1cm. The plate was gently moved to facilitate aggNET-formation after addition of cells ***(B)*** Human neutrophils developed web-like extracellular DNA (PI), when exposed to an isotonic saline, containing 75 mM $NaHCO_3$, as found in postprandial pancreatic juice, which shows ELANE ***(top)*** and H3cit positivity ***(bottom)***. ***(C-G)*** Quantitative kinetic assessment of DNA in human neutrophil cultures under ambient pCO_2 using Sytox Green, not permeant to live cells, indicates the rapid increase in DNA^{SYTOX} in response to $NaHCO_3$ (10-100 mM) ***(C)***, whereas no increase in DNA^{SYTOX} was observed in the absence of $NaHCO_3$. ***(D)*** Human pancreatic juice similarly induced an increase of DNA^{SYTOX}, comparable to a $NaHCO_3$-containing buffer (100 mM). PMA (100 nM) served as positive control for NETosis. **Table 1**: Clinical chemistry of the pancreatic juice sample analysed in Figure S8D. The range of each parameter in the set of samples analysed during the study can be found in the brackets. ***(E)*** Osmolality changes in the range theoretically induced by loss of 50 mOsm/kg HCO_3^- did not alter levels of DNA^{SYTOX}. ***(F)*** The threshold of DNA^{SYTOX} elevation due to alkaline pH was determined to be above pH 8.0 in bicarbonate-free phosphate-buffered solutions. ***(G)*** Increases in DNA^{SYTOX} induced by either $NaHCO_3$ (37 mM) or pancreatic juice devoid of tryptic activity were strongly attenuated by the carbonic-anhydrase-inhibitor acetazolamide as compared to solvent DMSO control. ***(H, I)*** Human neutrophils preincubated in the presence or absence of the PADI inhibitor Cl-amidine (1 mM) or respective DMSO control were cultured at 37°C / 5%CO_2 in isotonic 50 mM $NaHCO_3$-containing HBSS. ***(H)*** Note the reduced areas of individual nuclei in Cl-amidine-treated granulocytes. ***(I)*** A quantitative analysis of fluorescence intensity and area demonstrated the significant reduction of $Area^{hi}MFI^{lo}$ NETs / aggNETs in the presence of Cl-amidine. ***(J)*** Bicarbonate and $CaCO_3$-induced aggNETs derived from neutrophils (10^7 cells/ml) were subjected to DNase digestion (100 µg/ml) for 2 hours. Fine chromatin tangles were readily digested, a large aggNET resisted longer. Also note the carbonate-crystals (appearing black), only visible after DNase digestion. Black scale bars represent 200 µm, white bars 50 µm, unless stated otherwise. ***: $p<0.001$, Student's t-test.

Supplementary Figure 9 (Movies)

A

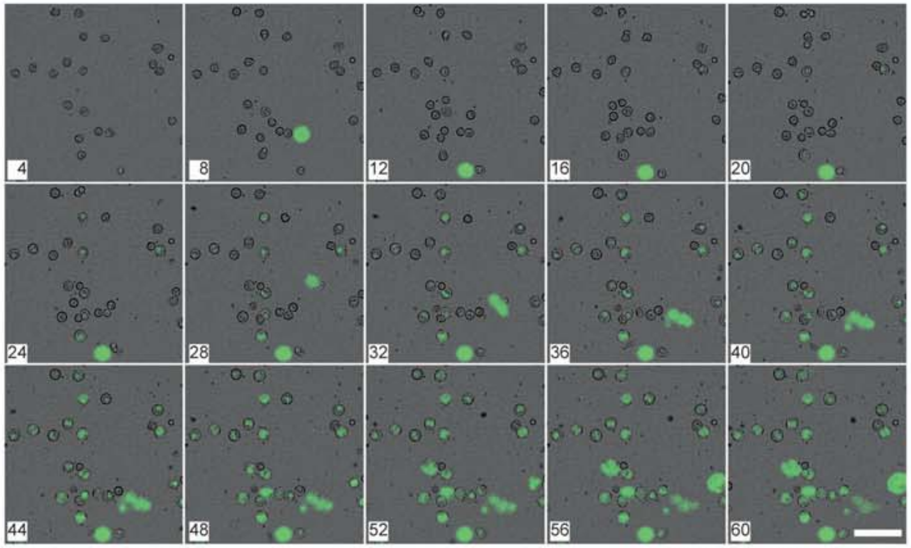

Human Pancreatic Juice / Chronic Pancreatitis (interval: 4 min; total: 60 minutes)

extracellular DNA: Sytox Green

C

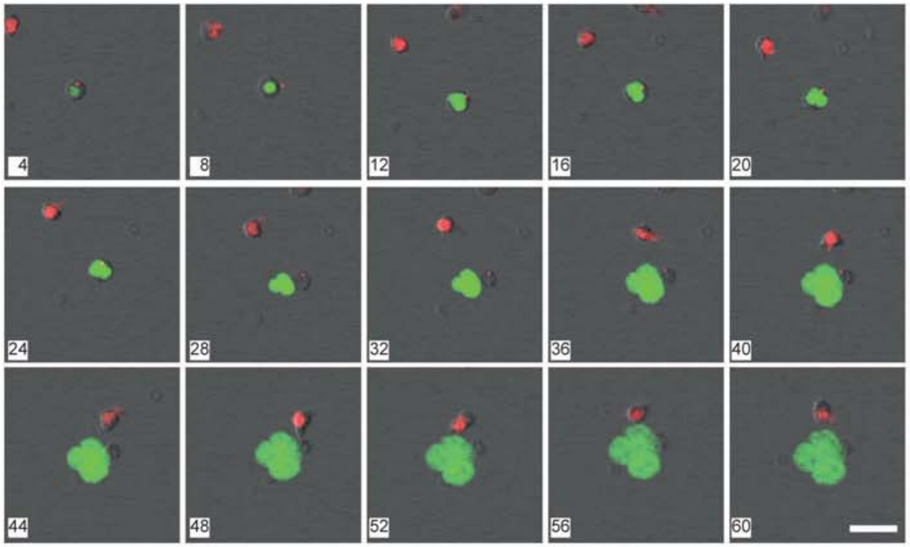

50 mM $NaHCO_3$ (interval: 4 min; total: 60 minutes)

pH: pHrodo / extracellular DNA: Sytox Green

B

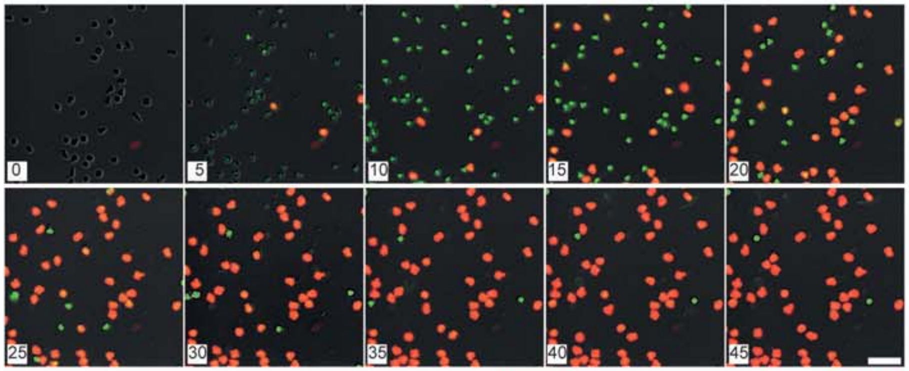

150 mM $NaHCO_3$ (interval: 5 min; total: 45 minutes)

DNA: Hoechst 33342 / extracellular DNA: propidium iodide

D

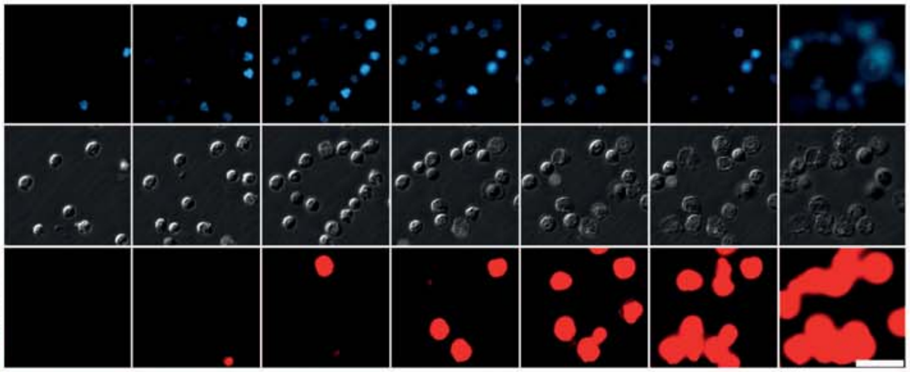

150 mM $NaHCO_3$ (interval: 150 s; total: 15 minutes)

Supplementary Figure 9: $NaHCO_3$ and pancreatic juice induce extrusion of chromatin in human granulocytes.

(Time-lapse microscopy is contributed as movie files for online use and as still movies in Supp. Figure 9)

(A, Movie 1) Human neutrophils underwent chromatin decondensation and rapid extrusion, when cultured in human pancreatic juice at 37°C / 5% CO_2 (50% isotonic juice, 50% HBSS isotonic medium). ***(B, Movie 2)*** Human PMN subjected to 150 mM $NaHCO_3$ supplemented with 5% FCS underwent morphological changes and forcefully released DNA. Viable PMN showed low propidium iodide (red) and high Hoechst 33342 signal (green). ***(C, Movie 3)*** Human neutrophils loaded with an intracellular pH dye (pHrodo), which exhibits red fluorescence only at acidic pH, were cultured at 37°C / 5% CO_2 in isotonic HBSS supplemented with 50 mM $NaHCO_3$ in the presence of the non-cell permeant DNA probe Sytox Green. A neutrophil already displaying alkalinized cytoplasmic pH executed sudden DNA release, whereas an adjacent cell, which has still retained its neutral pH did not. A similar effect was also observed in other cells of the cultures. ***(D, Movie 4)*** Human PMN underwent cellular spreading and disintegration (notable in differential interference contrast [DIC]), chromatin decondensation (visualized by Hoechst 33342 [blue]) and DNA extrusion (visualized by propidium iodide [red]) in response to 150 mM $NaHCO_3$ supplemented with 5% FCS. Externalized DNA of densely packed PMN leads to aggNET formation.All white scale bars are 50µm, unless otherwise stated. (Movie 5 provided as online movie file only).
